# Effect of oxic and anoxic conditions on intracellular storage of polyhydroxyalkanoate and polyphosphate in *Magnetospirillum magneticum* strain AMB-1

**DOI:** 10.3389/fmicb.2023.1203805

**Published:** 2023-06-15

**Authors:** Qingxian Su, Henrik Rasmus Andersen, Dennis A. Bazylinski, Marlene Mark Jensen

**Affiliations:** ^1^Department of Environmental and Resource Engineering, Technical University of Denmark, Lyngby, Denmark; ^2^School of Life Sciences, University of Nevada at Las Vegas, Las Vegas, NV, United States

**Keywords:** magnetotactic bacteria, oxygen, polyhydroxyalkanoate, polyphosphate, intracellular inclusion

## Abstract

Magnetotactic bacteria (MTB) are microorganisms widely inhabiting the oxic-anoxic interface of aquatic environments. Beside biomineralizing magnetic nanocrystals, MTBs are able to sequester various chemical elements (e.g., carbon and phosphorus) for the biogenesis of intracellular granules, like polyhydroxyalkanoate (PHA) and polyphosphate (polyP), making them potentially important in biogeochemical cycling. Yet, the environmental controls of intracellular storage of carbon and phosphorus in MTB remain poorly understood. Here, we investigated the influence of oxic, anoxic and transient oxic-anoxic conditions on intracellular storage of PHA and polyP in *Magnetospirillum magneticum* strain AMB-1. In the incubations with oxygen, transmission electron microscopy revealed intercellular granules highly rich in carbon and phosphorus, which were further interpreted as PHA and polyP based on chemical and Energy-Dispersive X-ray spectroscopy analysis. Oxygen had a strong effect on PHA and polyP storage in AMB-1 cells, as PHA and polyP granules accounted for up to 47 ± 23% and 5.1 ± 1.7% of the cytoplasmic space, respectively, during continuous oxic conditions, while granules disappeared in anoxic incubations. Poly 3-hydroxybutyrate (PHB) and poly 3-hydroxyvalerate (PHV) accounted for 0.59 ± 0.66% and 0.0033 ± 0.0088% of dry cell weight, respectively, in anoxic incubations, while the values increased by a factor of 7 and 37 after oxygen was introduced. The results highlight a tight link between oxygen, carbon and phosphorus metabolisms in MTB, where favorable oxic growth conditions can lead to metabolic induction of polyP and PHA granule biogenesis.

## 1. Introduction

Many microorganisms can form intracellular inclusions by sequestering various chemical elements (e.g., carbon, phosphorus, and sulfur) from the aquatic surroundings into relatively stable solid phases (Mino et al., 1998; Shively, 2006; Benzerara et al., 2011; Maki, 2013). The known chemical composition and physiological function of inclusions vary depending on the group of microorganisms forming them. Among the different intracellular inclusions are polyhydroxyalkanoates (PHAs), which mainly include poly 3-hydroxybutyrate (PHB) and poly 3-hydroxyvalerate (PHV). These polyesters are produced in microbial cells as carbon and energy storage compounds and electron sinks (Shively, 2006; Jendrossek, 2009). Another storage compound is polyphosphate (polyP), which is a linear polymer of tens to hundreds of phosphate residues linked together by high-energy bonds. The biological function of polyP granules differs widely among microorganisms, including inorganic phosphate storage reservoir, energy source, cation chelation, and environmental stress buffer (Kornberg, 1995; Albi and Serrano, 2016; Akbari et al., 2021). Typical examples of PHA-and/or polyP-containing microbes include polyphosphate-accumulating organisms (PAO) predominantly associated with enhanced biological phosphate removal (EBPR) in wastewater treatment plants, and sulfide oxidizing bacteria that are ubiquitous in marine sediments. The availability of oxygen (O_2_) in addition to carbon, nitrogen, and sulfur compounds have all been shown to drive PHA and polyP synthesis and consumption in bacteria (Gray and Jakob, 2015; Blunt et al., 2018). For instance, under alternating oxic and anoxic conditions in engineered EBPR systems, PAOs store polyP under oxic conditions by degrading PHA as energy source, thus removing phosphorus from wastewaters (Mino et al., 1998). Also filamentous sulfur bacteria within the family *Beggiatoaceae*, which are abundant in marine sediments with oscillating redox conditions, accumulate high amounts of inorganic phosphate and store it as polyP (Brock and Schulz-Vogt, 2011; Brock et al., 2012). Incubations with the model organism-marine *Beggiatoa* strain (35Flor *Beggiatoa*) under defined redox conditions suggested an extensive accumulation of polyP in *Beggiatoa* filaments under oxic conditions, while anoxic conditions with increasing sulfide concentrations led to a decomposition of polyP (Brock and Schulz-Vogt, 2011). The intracellular metabolisms of phosphorus and accumulation/release of polyP associated with redox changes remain unknown.

Magnetotactic bacteria (MTB) synthesize intracellular iron-rich inclusions called magnetosomes. They consist of magnetic nanocrystals in the form of magnetite (Fe_3_O_4_) and/or greigite (Fe_3_S_4_) enveloped by a lipid bilayer membrane (Bazylinski et al., 1995; Bazylinski and Frankel, 2004; Schüler, 2008). Magnetosomes enable MTB to align with the Earth’s magnetic field lines allowing MTB to navigate more efficiently across preferred environmental niches (Frankel et al., 1997). MTB are cosmopolitan in distribution and ubiquitous in both freshwater and marine systems. While some cultivated species are obligate anaerobes (e.g., *Desulfovibrio magneticus* strain RS-1), most of the reported MTB are microaerophiles (Lefevre and Bazylinski, 2013). The microaerophilic lifestyle of most MTB allows them to inhabit the oxic-anoxic interface of sediments and water columns (Bazylinski and Frankel, 2004). In fact, the highest numbers of MTB were observed at the oxic-anoxic interface of stratified water columns (Moskowitz et al., 2008). Electron microscopic images revealed that cultured and uncultured MTB contained other intracellular inclusions than magnetosomes, such as granules of PHA, polyP, sulfur and calcium carbonate (Lins and Farina, 1999; Cox et al., 2002; Keim et al., 2005; Silva et al., 2008; Lefèvre et al., 2009; Rivas-Lamelo et al., 2017; Schulz-Vogt et al., 2019; Li et al., 2020, 2021; Monteil et al., 2021; Goswami et al., 2022). Due to the metabolic versatility of MTB and their miscellaneous intracellular inclusions, they might play an important role in the biogeochemical elemental cycling of iron, carbon, phosphorus and sulfur. For instance, the large magnetotactic cocci with phosphorus rich inclusions were suspected to contribute significantly to the phosphorus cycling in stratified water column of Black Sea (Schulz-Vogt et al., 2019). Based on increased gene expression of polyP kinases (*ppk1* and *ppk2*) at the phosphate maximum, MTB within the genus *Magnetococcus* were hypothesized to take up phosphate at the upper boundary of suboxic zone and release it again at the lower boundary (Schulz-Vogt et al., 2019). However, the storage capacity of PHA and polyP in MTB, and the influence of redox conditions on the synthesis and decomposition of PHA and polyP have so far not been investigated in controlled laboratory incubations. Understanding the accumulation and release pattern of intracellular inclusions of cultivated MTB under manipulated redox conditions may provide an important framework for discerning their potential biogeochemical roles in natural environments.

In the well-studied MTB *Magnetospirillum magneticum* strain AMB-1, cells were frequently observed to contain PHA and polyP granules visualized with scanning transmission electron microscopy (STEM; Olszewska-Widdrat et al., 2019; Amor et al., 2020; Wan et al., 2022). Stain AMB-1, originally isolated from freshwater sediment, is a facultative anaerobe and thus capable of growing aerobically, and is abundantly distributed at the surface of sediments (Matsunaga et al., 1991). The main objective of this study was to systematically assess the effect of oxic, anoxic, and transient oxic-anoxic conditions on intracellular storage of PHA and polyP in strain AMB-1. Batch incubations were performed in parallel by exposing cells of AMB-1 to different oxic and anoxic regimes. To characterize the effect of O_2_ on the accumulation and release of PHA and polyP, inclusions were examined through STEM combined with Energy-Dispersive X-ray spectroscopy (EDX), and the concentration and composition of PHA were further analyzed through chemical analysis. Finally, the potential mechanisms underlying PHA and polyP storage in AMB-1 were proposed.

## 2. Materials and methods

### 2.1. Cultivation conditions

We focused on the MTB *Magnetospirillum magneticum* strain AMB-1 in this study. Cells were grown in 60 mL of liquid medium in 100 mL serum bottles at 25°C. Anoxic growth media contained: 5 mL Wolfe’s mineral solution, 0.68 g potassium dihydrogen phosphate (KH_2_PO_4_), 0.51 g sodium succinate anhydrous (C_4_H_4_Na_2_O), 0.58 g sodium tartrate dibasic dihydrate (C_4_H_4_Na_2_O_6_·2H_2_O), 0.050 g sodium acetate anhydrous (C_2_H_3_NaO_2_), 0.17 g sodium nitrate (NaNO_3_), 0.040 g ascorbic acid (C_6_H_8_O_6_), 3 mL 10 mM ferrous sulfate (FeSO_4_), 200 μL 0.2% resazurin and 0.5 mL vitamin solution per liter Milli-Q water. The detailed compositions of Wolfe’s mineral solution and vitamin solution were described in Wolin et al. (1963) and the website of the Deutsche Sammlung von Mikroorganismen und Zellkulturen (DSMZ) (Medium 141), respectively. All chemicals were purchased from Sigma-Aldrich (Germany). The pH of the medium was adjusted to 6.75–7.00, and O_2_ in the headspace was adjusted to ~2%.

### 2.2. Oxic and anoxic batch incubations

To investigate the effect of the oxygen regime on PHA and polyP inclusions in AMB-1, the pre-cultivated AMB-1 cells were incubated under oxic, anoxic and transient oxic-anoxic conditions (Supplementary Figure S1). The 600 mL serum bottles contained two-thirds volume of anoxic sterile growth media in which ascorbic acid and resazurin were omitted. Bottles were closed with butyl rubber stoppers and the headspace was flushed with dinitrogen gas. AMB-1 cells were harvested in the middle of the exponential growth phase and centrifuged. The concentrated AMB-1 cells were subsequently inoculated into the serum bottles, resulting in the initial optical density at 565 nm (OD_565_) of 0.013 ± 0.0019. In parallel incubations, AMB-1 cells were subject to four different oxic and anoxic regimes for 20 days (Supplementary Table S1; Supplementary Figure S1). The incubation periods were named after the oxygen regime and incubation days (Supplementary Table S1). For example, 20 days of incubation under constant oxic conditions was named O20. In comparison, incubation under intermittent oxic-anoxic conditions was named O7A5O8 under 7 days of oxic condition followed by 5 days of anoxic condition and another 5 days of oxic condition again. The anoxic conditions were achieved by flushing the headspace with nitrogen gas (N_2_), while pure O_2_ was added to provide oxic conditions. O_2_ concentrations in the media were manually controlled by monitoring with PyroScience noninvasive optical oxygen sensors (Germany) and injection of pure O_2_. As AMB-1 is typically grown with a low O_2_ concentration (2–10%) in the culture headspace (McCausland et al., 2021), O_2_ concentrations were controlled at 5.1 ± 1.2 μM during oxic conditions (n = 50). During anoxic conditions, O_2_ concentrations were below the detection limit of the oxygen sensors (~0.0052 μM) (data not shown). The bottles were incubated at 25°C with gentle shaking at 100 rpm in the dark. Cell growth was determined daily by measuring OD_565_. The correlation between OD_565_ values and cell number was determined by cell-counting using Thoma cell counting chamber (ThermoFisher Scientific, United States) with an optical microscope (Zeiss, Germany). The linear relationship of OD_565_ with cell density was given by function: Cell density = 3.0 × 10^8^·OD_565_ + 1.0 × 10^6^ (*R*^2^ = 0.995). An OD_565_ of 0.1 corresponded to a cell density of 3.1 × 10^7^ cells/mL, which was similar to the value (i.e., 3.3 × 10^7^ cells/mL) calculated in a previous study using the cell counting method for *Magnetospirillum* strains (Heyen and Schüler, 2003). Culture samples (~25 mL) were collected daily for STEM visualization and various chemical analyses.

### 2.3. Scanning transmission electron microscopy and energy dispersive X-ray spectroscopy analysis

The harvested cells from the different incubations were washed three times with Milli-Q water and concentrated by centrifugation at 2,500 rpm for 5 min. Approximately 10 μL of washed cells was loaded onto a 200-mesh Formvar–carbon-coated copper grid (Agar Scientific). The cells were allowed to settle for 15 min, and water was carefully removed with filter paper (Whatman, Germany). The grids were left to dry before microscopic analysis. Cell visualization and elemental analysis were conducted at STEM mode of Fei Quanta FEG 200 ESEM equipped with an Oxford Instruments EDX spectrometer. The electron microscope was operated at high voltage of 10–30 kV and a working distance of 10 mm. Area and diameter of AMB-1 cells and inclusions were measured from STEM images using ImageJ software (1.53v).

### 2.4. Analytical measurements

For solid-phase PHA extraction, 20 mL of AMB-1 cultures were centrifuged at 4,000 rpm for 10 min at 4°C. The cell debris were stored at −18°C until PHA extraction, while the supernatant was filtered through 0.22 μm-pore-diameter polypropylene filters for analyses of nitrogen species, phosphate (PO_4_^3−^), Fe^2+^ and organic carbon (i.e., sodium succinate, sodium tartrate and sodium acetate).

PHAs were extracted by a modified version of the sodium hypochlorite (NaClO) digestion method (Hierro-Iglesias et al., 2021). Prior to the extraction, AMB-1 cells were freeze-dried for 24 h. Weighed freeze-dried cells (~7 mg) were suspended in 2 mL of 13% NaClO (w/v; pH = 11.8), and incubated at room temperature. After 1 h, 2 mL of milli-Q water was added to enhance the PHA sedimentation rate, followed by another 8 h of incubation at room temperature. After centrifugation at 4000 rpm for 10 min, the supernatant (containing water-soluble components) was removed, while the pellet (containing PHA) was resuspended in 2 mL of 70% isopropanol (w/v). After freeze-drying overnight, cell debris was weighed and digested at 100°C in 2 mL of acidified methanol (20% sulfuric acid v/v) and 1 mL of chloroform (containing an exact amount of heptadecane (*ca.* 1 g/L) as internal standard) for 3.5 h. After incubation, 1 mL water was added to enhance phase separation. The lower phase containing PHA was transferred into 2 mL glass vial. Samples were analyzed by gas chromatography equipped with a flame-ionization detector and a column (60 m, 0.53 mm internal diameter, 1 μm film thickness) coupled with a guard-column (0.32 mm internal diameter). Helium was utilized as carrier gas at constant pressure (14.5 psi), and the temperature of injection and detector was 280°C and 230°C, respectively. PHB and PHV were the two target compositions of PHA to be analyzed. The compound was confirmed by retention time and mass spectral matching with known PHA standards (a commercial co-polymer of PHB-PHV (88:12 M)), and quantified based on the internal standard.

An air-segmented continuous-flow analyzer (SKALAR San^++^, Netherlands) was used for colorimetric analysis of ammonium (NH_4_^+^), nitrite (NO_2_^−^), NO_x_^−^ (i.e., NO_2_^−^ + nitrate (NO_3_^−^)) and PO_4_^3−^. Dissolved reduced iron (Fe^2+^) was determined colorimetrically using a Ferrozine solution (50 mmol/L HEPES, 0.08% Ferrozine, pH 7) (Stookey, 1970; Thamdrup et al., 1994). Organic carbon concentrations of sodium succinate anhydrous, sodium tartrate and sodium acetate were analyzed by high performance liquid chromatography (HPLC) with a refractive index detector. pH was monitored by a pH probe (WTW GmbH, Weilheim, Germany).

## 3. Results and discussions

### 3.1. Growth of and magnetosome formation in AMB-1 cells

**Figure 1:**
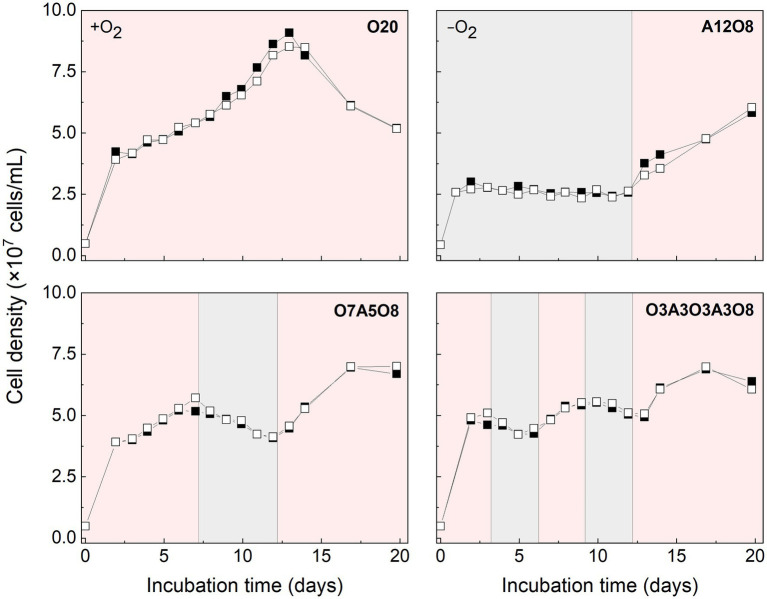
Cell density of AMB-1 cultures during incubation period. Pink and grey colors indicate oxic and anoxic periods, respectively. Black and white squares define parallel incubations. The different incubation periods were named after oxygen regime and incubation days (Supplementary Table S1).

Growth of AMB-1 and magnetosome formation were influenced by O_2_ (Figure 1). Transient high growth rates were observed within the first 48 h, i.e., (2.0 ± 0.21) × 10^7^ cells/mL/d and (1.2 ± 0.075) × 10^7^ cells/mL/d under oxic and anoxic conditions, respectively (Figure 1). Fast growth was accompanied by a decrease in the concentrations of sodium succinate, sodium tartrate, NO_3_^−^, PO_4_^3−^ and dissolved Fe^2+^ (*p* < 0.05; Supplementary Figures S2–S5). After 48 h, AMB-1 cell density continued to increase in the presence of O_2_, while anoxic conditions resulted in stagnated growth or a net loss of cells (Figure 1). As facultative anaerobic bacteria, AMB-1 can use NO_3_^−^ as the terminal electron acceptor during growth under anoxic conditions, while respiratory oxygen reduction was suggested to facilitate growth under low O_2_ conditions (Yang et al., 2001; Heyen and Schüler, 2003; Matsunaga et al., 2005; Olszewska-Widdrat et al., 2019). Based on complete genome sequence, AMB-1 has been shown to possess genes (including nitrate reductase, nitrite reductase, nitric oxide reductase, and nitrous oxide reductase) for a complete denitrification pathway (Matsunaga et al., 2005). However, strict anaerobic conditions have been reported to inhibit AMB-1 growth (Yang et al., 2001; Heyen and Schüler, 2003). Similar to the results in this study, cell density was observed to decrease by half when changing initial O_2_ concentrations from 3.9 to 0% in previous AMB-1 batch incubations (Popa et al., 2009).

**Figure 2:**
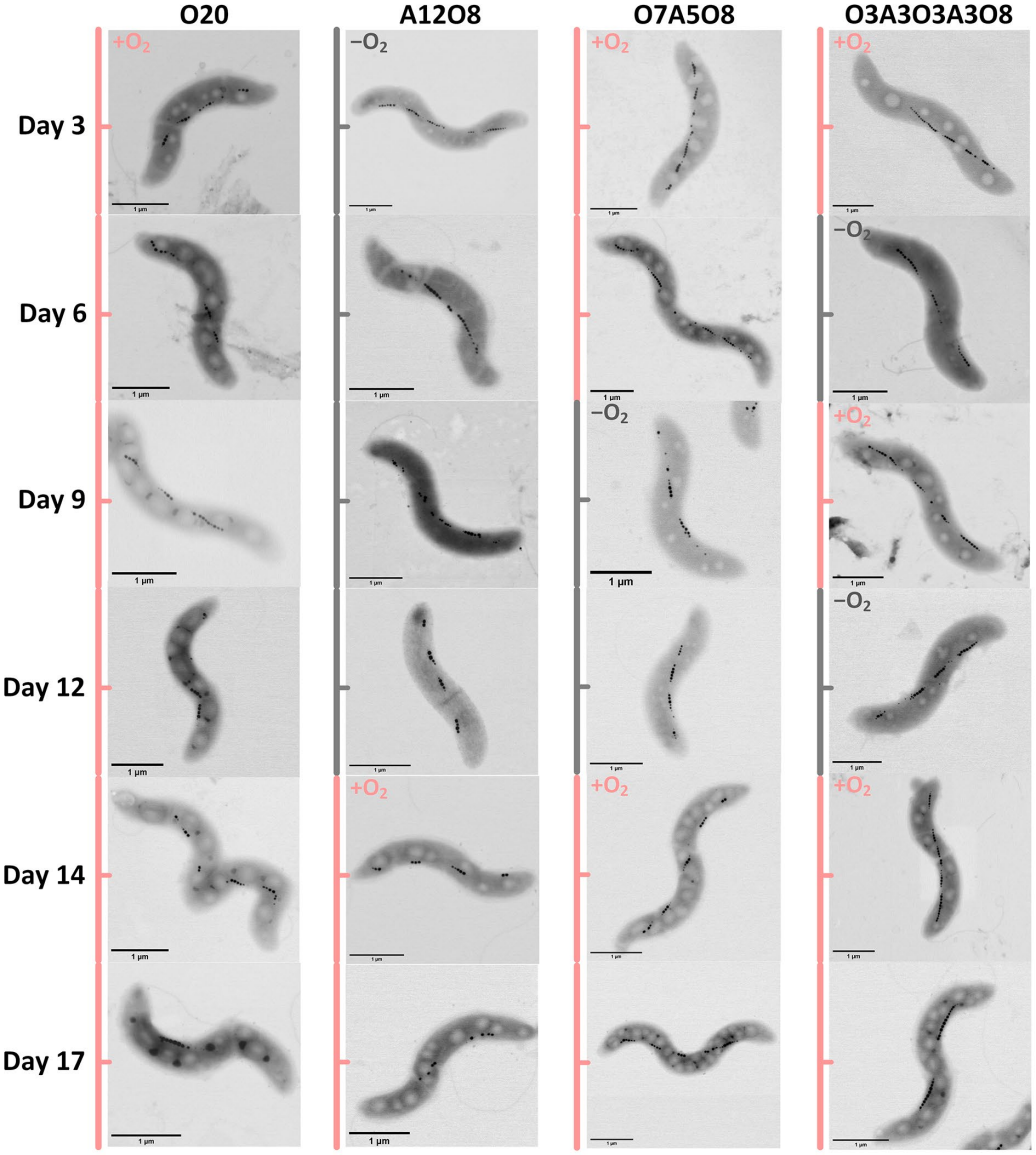
Examples of STEM images of AMB-1 cells at different incubation time. Pink and grey colors indicate oxic and anoxic periods, respectively. The different incubation periods were named after oxygen regime and incubation days (Supplementary Table S1).

**Figure 3:**
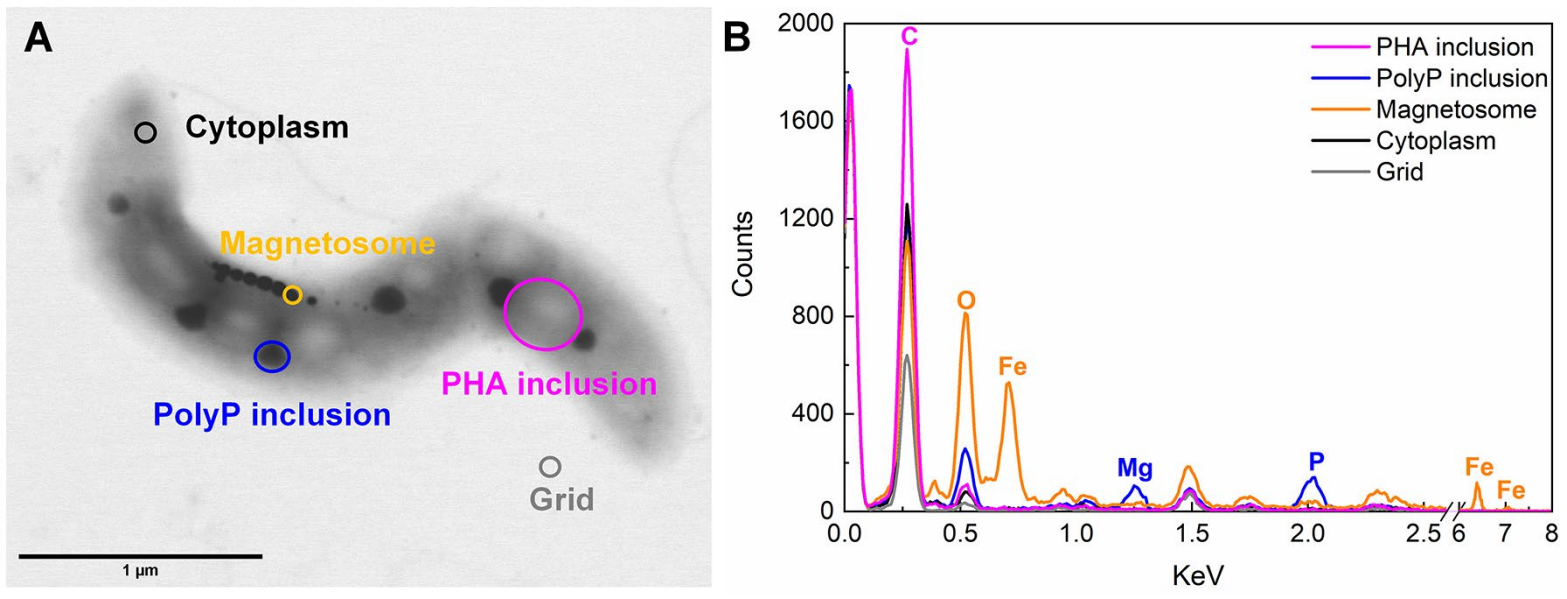
Example of STEM-EDX illustration **(A)** and analysis **(B)** of inclusions in AMB-1 (day 17).

Based on STEM images (Figures 2, 3), AMB-1 cells showed a spirillum morphology with a mean area of 1.8 ± 0.29 μm^2^ (4.3 ± 1.6 μm in length and 0.56 ± 0.095 μm in width; *n* = 277). AMB-1 cells contained 10–47 magnetosomes in 1–5 bundles of multiple chains (Figures 2, 3). Also, EDX analysis of magnetosomes showed high content of iron and oxygen (Figure 3), suggesting that magnetite (Fe_3_O_4_) was the major component of magnetosomes of AMB-1. Similar diameter (41 ± 10 nm) and size [(1.7 ± 0.84) × 10^−3^ μm^2^] of magnetosomes were obtained during different imposed conditions in our AMB-1 batch incubations. There was no significant change (*p* > 0.05) in magnetosome number per cell during short intervals of oxic-anoxic transition, such as during O3A3O3A3O8 and O7A5O8 incubations (Supplementary Figure S6). In contrast, fewer magnetosomes (per cell) were formed after long anoxic periods, gradually decreasing from 22 ± 5 at day 3 to 15 ± 3 at day 12 for A12O8 incubation, compared to continuous oxic incubations (e.g., averaged 23 ± 3 during O20 incubation; *p* < 0.05). While too high O_2_ and anoxic conditions would result in lower magnetosome formation, a low O_2_ concentration of 2–8 μM was found optimal for magnetosome formation of AMB-1 (Yang et al., 2001; Heyen and Schüler, 2003; Ge et al., 2011; Li and Pan, 2012; Olszewska-Widdrat et al., 2019). As the oxygen in MTB-biomineralized magnetite comes from water based on previous isotope analysis (Mandernack et al., 1999), the formation of magnetosomes thus seemed indirectly dependent on dissolved O_2_ concentration. Although the underlying molecular mechanism remains unclear, our oxygen-shift experiments indicated that O_2_ likely served as regulatory signal for metabolic induction of the biomineralization of magnetosomes.

### 3.2. Intracellular storage of carbon and phosphorus in AMB-1

Apart from magnetosomes, AMB-1 cells also contained two additional types of electron-dense inclusions: large electron-lucent and small dark ellipse-shape granules (Figures 2, 3). White and black inclusions were 0.25 ± 0.090 μm and 0.084 ± 0.031 μm in diameter, and 0.049 ± 0.030 μm^2^ and 0.0053 ± 0.0038 μm^2^ in area, respectively (*n* = 777–2,138). Compared to the cytoplasmic background, EDX analysis of white inclusions indicated a high content of carbon. A major carbon peak could indicate organic compounds in the granules. Conversely, the black inclusion contained mainly phosphorus together with oxygen and magnesium (Figure 3).

#### 3.2.1. Effect of oxic and anoxic conditions on PHA storage

**Figure 4:**
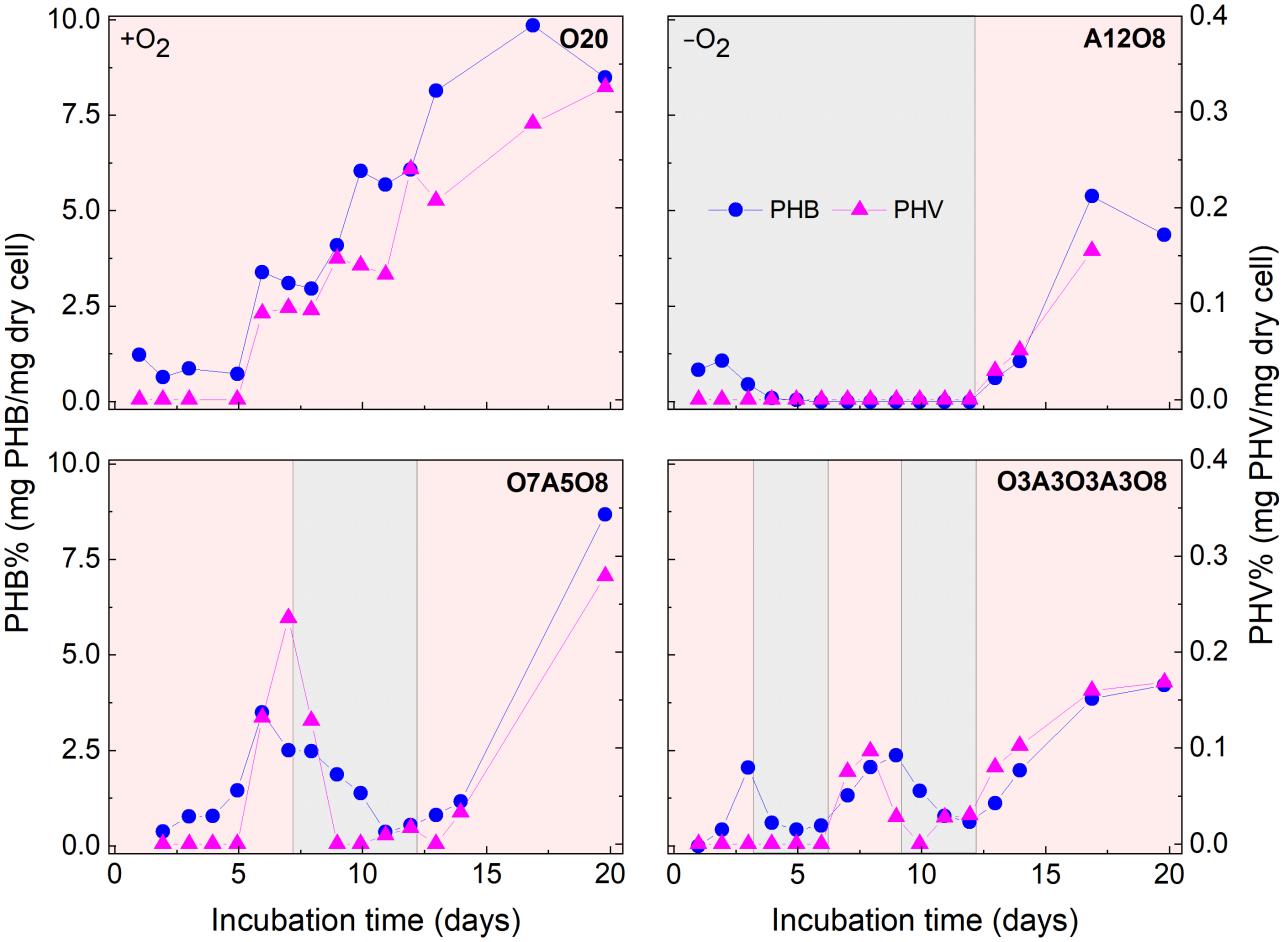
Changes in poly 3-hydroxybutyrate (PHB) and poly 3-hydroxyvalerate (PHV) content in AMB-1 cells during incubation period. Pink and grey colors indicate oxic and anoxic period, respectively. The different incubation periods were named after oxygen regime and incubation days (Supplementary Table S1).

**Figure 5:**
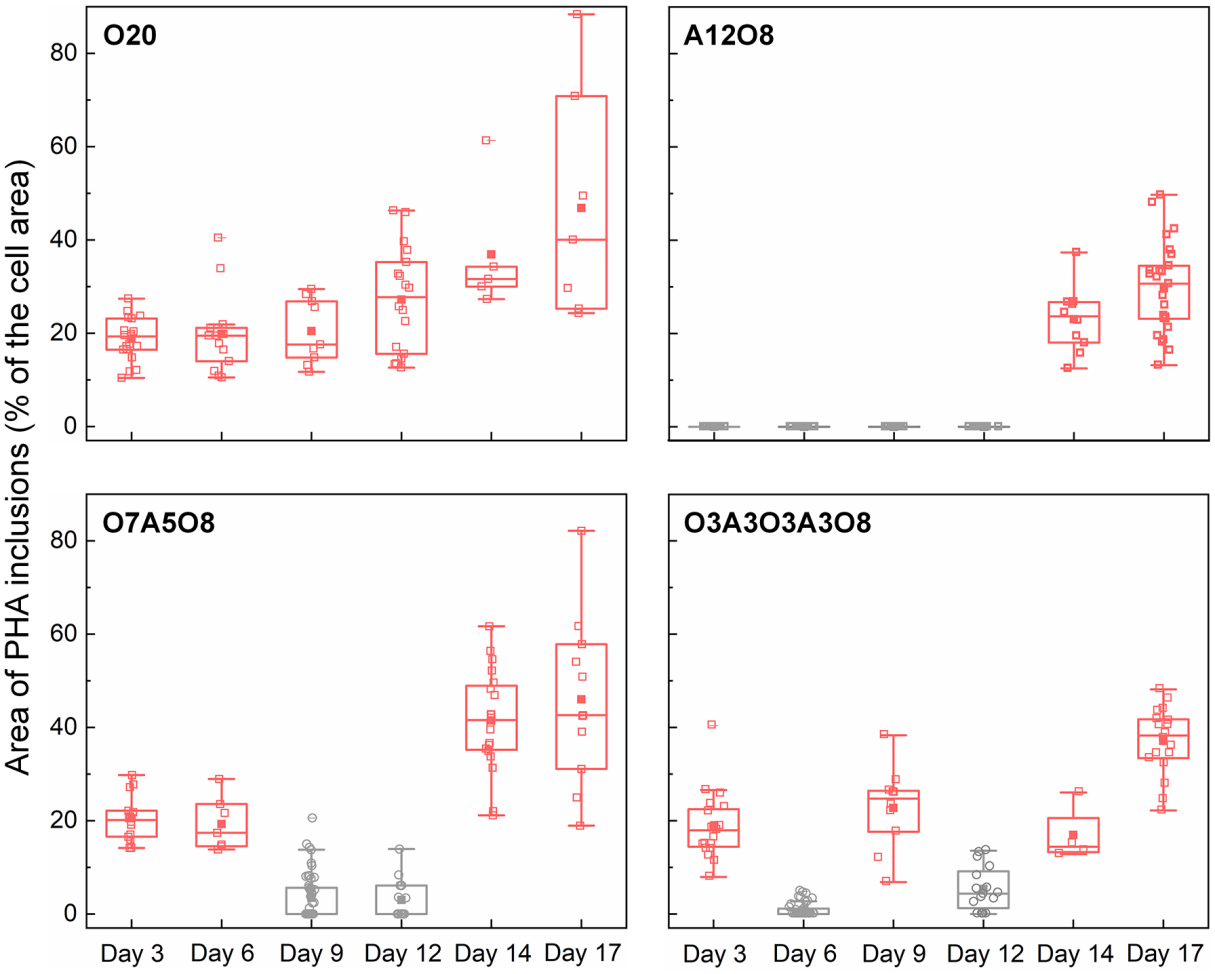
Changes in the area of PHA inclusions during incubation period (n = 43–212 cells). The area of PHA inclusions (% of the cell area) was calculated by dividing the sum of the area of PHA inclusions (μm^2^) in an AMB-1 cell by the area of the cell (μm^2^). The number, diameter, and area of PHA inclusions are presented in Supplementary Figures S7–S9. Pink and grey colors indicate oxic and anoxic period, respectively. The different incubation periods were named after oxygen regime and incubation days (Supplementary Table S1).

There were consistent trends of white inclusions observed in STEM images and PHA concentrations analyzed through chemical analysis under different oxic and anoxic regimes, i.e., more white inclusions and higher PHA concentrations under oxic conditions and vice versa under anoxic conditions (Figures 2, 4, 5). Combined with EDX analysis, the results indicated that the carbon containing inclusions were highly PHA-rich. Intracellular PHA granules in bacteria occur mostly as different types of homo-and heteropolymers (i.e., PHB), and/or poly (3-hydroxybutyric-co-3-hydroxyvaleric acid) [P(3HB-co-3 HV)] (Khanna and Srivastava, 2005; García et al., 2013; Hierro-Iglesias et al., 2021). During oxic conditions in our different incubations, analysis of extracted PHA revealed the presence of PHB copolymerized with PHV in AMB-1, with the averaged ratio of PHB/PHV at (98 ± 1.9):(2.1 ± 1.7) (mol%; *n* = 160) (Figure 4). PHA aggregates have previously been reported in different MTB, such as *Magnetospirillum gryphiswaldense* strain MSR-1, *Magnetococcus marinus* strain MC-1, and *Candidatus* Magnetoglobus multicellularis, using chemical analytical and staining methods (e.g., Nile red staining; Schultheiss et al., 2005; Silva et al., 2008; Lefèvre et al., 2009; Fernández-Castané et al., 2017; Hierro-Iglesias et al., 2021). The calculated PHB/PHV ratio in our study was close to the value of 99:1 reported for MSR-1 in the exponential phase, which was grown under similar cultivation conditions (Hierro-Iglesias et al., 2021).

Referring to the carbon utilization by other PHA-accumulating microbes (Lemos et al., 1998; Lütke-Eversloh and Steinbüchel, 1999; Steinbüchel and Lütke-Eversloh, 2003), the potential carbon source of PHA synthesis for AMB-1 could be succinate, tartrate and acetate in medium (Supplementary Figure S2). These organics are converted to common intermediates, like acetyl-CoA that can be used as the substrate of PHA synthesis (Steinbüchel and Lütke-Eversloh, 2003). PHA yield was calculated as the unit amount of PHA produced (mmolC PHA) divided with unit amount of substrate consumed (mmolC carbon). For AMB-1 in our study, the estimated PHA yield was 0.036 ± 0.030 (maximum value of 0.11) under oxic conditions in all incubations, indicating that organic carbon added to medium was primarily used for cell growth (Figure 1; Supplementary Figure S2). The PHA yield was in the low range of values reported for other PHA-accumulating microbes (Lemos et al., 1998; Serafim et al., 2004; Albuquerque et al., 2007; Fradinho et al., 2014; Queirós et al., 2014; Sciarria et al., 2018). For instance, using an inoculum from a laboratory-scale EBPR reactor fed with acetate, Serafim and coworksers obtained a PHA storage yield of 0.68 mmolC PHA/mmolC carbon (Serafim et al., 2004). Bacteria enriched from activated sludge were reported to use acetate and butyrate as carbon sources for PHA synthesis, resulting in a maximum PHA yield of 0.77 ± 0.18 mmolC PHA/mmolC carbon (Sciarria et al., 2018). Since PHA granules occupied up to 88% of the cell area under oxic conditions (Figures 2, 5), the PHA yield of AMB-1 seemed to be underestimated, probably due to low recovery efficiency of the applied extraction method (Madkour et al., 2013). Further comparison between different PHA extraction methods using cultured AMB-1 cells would be helpful to achieve a more precise quantification of PHA storage capability. After depletion of organic carbon after day 10 in O7A5O8 and O3A3O3A3O8, and after approximately day 13 in O20, PHA continued to accumulate under oxic conditions (Figure 4; Supplementary Figure S2). The additional carbon source might be from the cell decay during anoxic periods (days 8–12 in O7A5O8, days 10–12 in O3A3O3A3O8), where cell growth stagnates and even decreases (Figure 1). In the oxic incubation, additional carbon source could originate during the stationary/death phase (days 14–20 for O20) (Figure 1). More investigations are needed in order to demonstrate the carbon source for PHA synthesis under different growth phases.

Oxygen had a profound effect on PHA storage in AMB-1 cells. Overall, bigger PHA inclusions and higher content of intracellular PHA inclusion were observed in cells during oxic periods, compared to anoxic conditions (Figures 2, 5). During continuous oxic conditions in O20, the number and area of PHA storage granules per cell increased remarkably with time, occupying 47 ± 23% of the cytoplasmic space on day 17 (Figures 2, 5; Supplementary Figures S7–S9). Conversely, PHA inclusions were not found during anoxic periods in A12O8, where they appeared after introducing O_2_ to the bottle. Here, PHA inclusions accounted for 30 ± 10% of the cytoplasmic space on day 17 (Figure 5). Similar trends were also observed during short-term oxic-anoxic transitions in O7A5O8 and O3A3O3A3O8, where PHA inclusions accumulated in the presence of O_2_, and decreased in both size and number in the absence of O_2_ (Figures 2, 5; Supplementary Figures S7–S9). For instance, in O7A5O8, the area percentage of PHA inclusions decreased from 19 ± 5.2% on day 6 (oxic) to 3.0 ± 4.0% on day 12 (anoxic), and subsequently increased again to 41 ± 17% on day 17 (oxic) (Figure 5). The aforementioned microscopic observations were consistent with PHB and PHV results (Figures 2, 4, 5). During anoxic conditions, PHB and PHV accounted for 0.59 ± 0.66% and 0.0033 ± 0.0088% of dry cell weight, respectively, while values increased up to 9.9% (averaged 4.3 ± 2.5%) and 0.33% (averaged 0.14 ± 0.089%) of dry cell weight under oxic conditions.

The presence of PHA inclusions has been reported in many bacteria (Steinbüchel et al., 1992; Pötter and Steinbüchel, 2006). PHA granules can act as storage compounds of carbon and energy, which are required for the maintenance of metabolism and synthesis of cellular metabolites during starvation, in particular after growth resumes, as well as an electron sink into which excess of reducing power can be channeled (Wältermann and Steinbüchel, 2005; Pötter and Steinbüchel, 2006). Besides the primary storage function, PHA also can enhance robustness and survival of bacterial cells against environmental stress conditions, like under high or low temperature, freezing, oxidative and osmotic pressure, which is likely associated with their extraordinary architecture and biophysical properties (Obruca et al., 2020). It has been previously reported that the biosynthesis of PHA was promoted under imbalanced nutrient conditions, such as an excess of carbon source and electron donor and lack of another nutrient (e.g., nitrogen or sulfur) (Kessler and Witholt, 2001; García-Torreiro et al., 2016; Fernández-Castané et al., 2017). In our incubations, sodium succinate anhydrous, sodium tartrate and NO_3_^−^ became limited from day 3–12 (Supplementary Figures S2, S3). Similar PHA storage and release patterns were observed under different oxic-anoxic transition regimes throughout the whole incubation period, revealing that O_2_ had the strongest influence on PHA accumulation. The role of O_2_ on PHA synthesis differs among different microorganisms (Mino et al., 1998; Borah et al., 2002). Similar to AMB-1, the presence of O_2_ has been observed to enhance PHA accumulations by *Bacillus mycoides* (Borah et al., 2002), while PHA formations only took place after exhaustion of O_2_ in PAOs and glycogen-accumulating organisms (GAOs) in EBPR systems (Mino et al., 1998).

#### 3.2.2. Effect of oxic and anoxic conditions on polyP storage

The third type of granule identified inside AMB-1 cell was the phosphorus-rich granule, which was smaller and more electron dense than PHA granule, and less electron dense than the magnetosome. Each AMB-1 cell contained up to 12 spherical phosphorus-rich granules, with the diameter in the range of 0.00022–0.19 μm, while some cells contained no granules right in phosphorus (Figures 2, 3; Supplementary Figures S10, S11). Phosphorus-rich inclusions in single cells were located adjacent to the PHA inclusions. Elemental EDX analysis showed that these phosphorus-rich granules besides phosphorus contained oxygen and magnesium as major elements, and small amount of potassium and calcium (Figure 3). Dark red color was observed when cells were stained with toluidine blue (data now shown). Based on the dark red colour and the presense of metals in phosphorus-rich granules, we assumed that phosphorus-rich granules in the AMB-1 cells were consisted of polyP (Brock and Schulz-Vogt, 2011; Schulz-Vogt et al., 2019). The presence of similar phosphorus-rich inclusions has previously been observed in MTB with transmission electron imaging, especially in uncultured magnetotactic cocci (Lins and Farina, 1999; Cox et al., 2002; Keim et al., 2005; Lefèvre et al., 2009; Rivas-Lamelo et al., 2017; Schulz-Vogt et al., 2019; Li et al., 2021; Goswami et al., 2022). These phosphorus-rich inclusions were also classified as polyP. For example, the volume of polyP granules made up most of the cell volume of magnetotactic cocci in Lake Pavin, France (Rivas-Lamelo et al., 2017). Besides of phosphorus with a relative abundance of 65.9 ± 3.2%, the authors found magnesium, potassium and calcium elements associated with polyP granules in relative abundances of 22.8 ± 4%, 5.1 ± 3.1% and 6 ± 5.4%, respectively (Rivas-Lamelo et al., 2017). PolyP inclusions were also observed in MTB of the genus *Magnetococcus* in the suboxic zone of Black Sea (Schulz-Vogt et al., 2019). In the Black Sea MTB, the inclusions contained 26–34% phosphorus and 1–5% metals (e.g., iron and manganese) (Schulz-Vogt et al., 2019). PolyP is generelly composed of linear polymers of orthophosphate linked through high energy phosphoanhydride bonds (Kornberg, 1995; Cox et al., 2002). Each orthophosphate unit carries a monovalent negative charge at physiological pH, resulting in a large cation exchange capacity of polyP. The binding energy facilitates polyP to sequester Mg^2+^, Ca^2+^, K^+^ etc., (Reusch, 2000; Cox et al., 2002), consistent with the signals detected in EDX spectra of phosphorus-rich granules in AMB-1 cells (Figure 3). The binding of these metals leads to the high electron density of these polyP granules.

**Figure 6:**
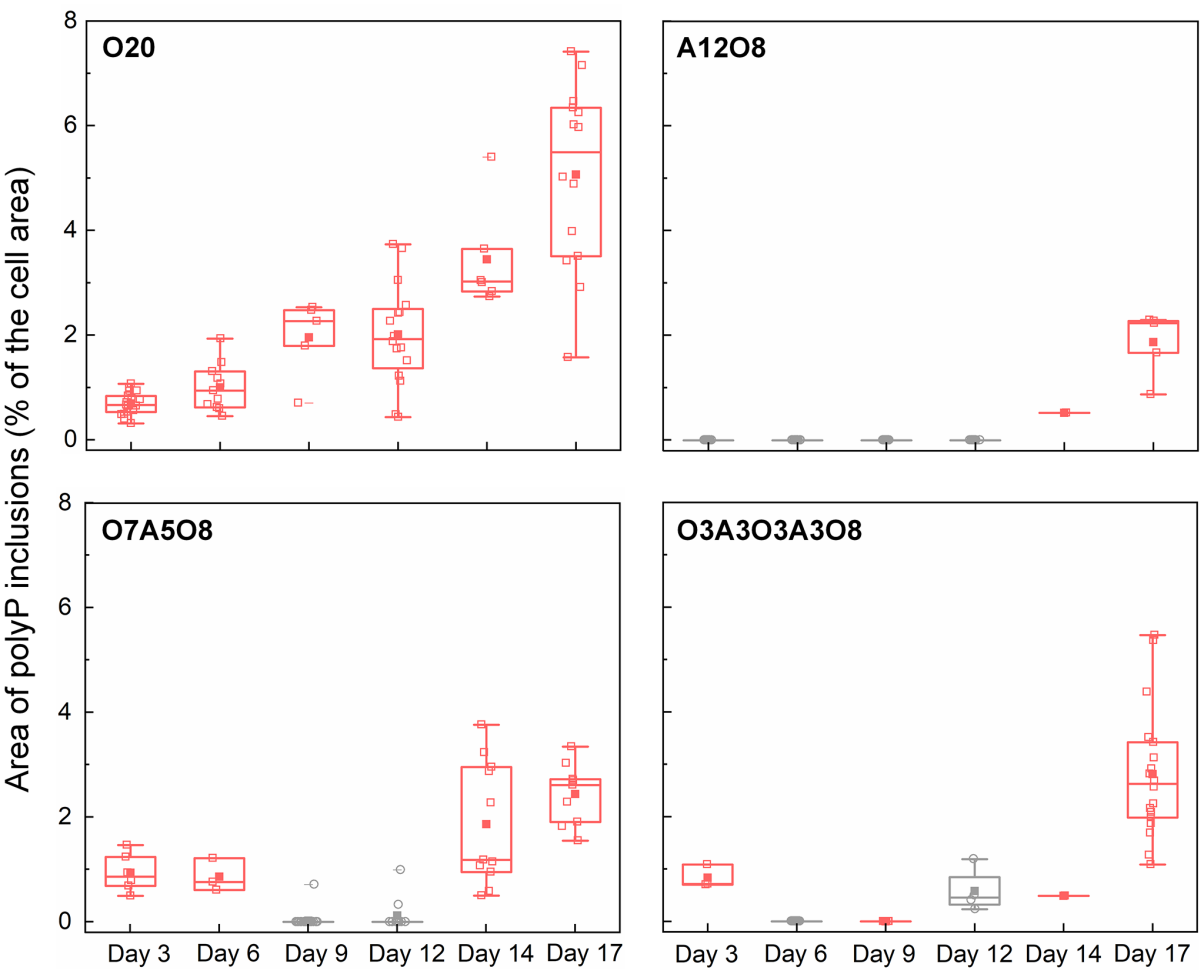
Changes in the area of polyP inclusions during incubation period (*n* = 27–114 cells). The area of polyP inclusions (% of the cell area) was calculated by dividing the sum of the area of polyP inclusions (μm^2^) in an AMB-1 cell by the area of the cell (μm^2^). The number, diameter, and area of polyP inclusions were presented in Supplementary Figures S10–S12. Pink and grey colors indicate oxic and anoxic period, respectively. The different incubation periods were named after oxygen regime and incubation days (Supplementary Table S1).

Similar to PHA inclusions, the presence of O_2_ strongly enhanced the intracellular storage of polyP in AMB-1 cells, with more and bigger polyP granules under oxic conditions (Figures 2, 6; Supplementary Figures S10–S12). During continuous oxic conditions in O20, the number and area of polyP granules gradually increased from 3.4 ± 1.6 and 0.0032 ± 0.0014 μm^2^ on day 3 to 6.5 ± 1.7 and 0.011 ± 0.0048 μm^2^ on day 17, respectively, accounting for 0.69 ± 0.21% (day 3) and 5.1 ± 1.7% (day 17) of the cytoplasmic space. Short O_2_ exposure time led to less polyP, with the area percentage of 1.9 ± 0.55% in A12O8, 2.4 ± 0.56% in O7A5O8, 2.8 ± 1.2% in O3A3O3A3O8 on day 17 (Figures 2, 6). PolyP granules were barely observed under anoxic conditions (Figures 2, 6). Similar to PHA synthesis, upon the depletion of PO_4_^3−^ in medium, any additional phosphorus source for polyP synthesis might originate from cell decay during anoxic periods where cell growth stagnates and even decreases (day 8–12 in O7A5O8, day 10–12 in O3A3O3A3O8), and during the stationary/death phase in the oxic incubation (day 14–20 for O20) (Figure 1; Supplementary Figure S5). We did not observe any release of PO_4_^3−^ after the transition from oxic to anoxic conditions. The amount of phosphorus released into medium might be too low to detect with the technique used in our study (detection limit of PO_4_^3−^ was ~0.058 mM). The phosphorus utilization for polyP synthesis under different growth phases and nutrient availability remains to be investigated in future studies.

The physiological function of polyP inclusions in MTB remains unclear. According to other non-MTB polyP-accumulating bacteria, such as PAO in EBPR reactors, these polyP inclusions might serve as a source of ATP, or a response to oxidative stress (Seviour et al., 2003). Rivas-Lamelo et al. (2017) speculated that the massive accumulation of polyP in magnetotactic cocci in Lake Pavin was due to the effect of oxic/anoxic fluctuations, either by travelling vertically over short distances from the anoxic to oxic zone (and vice versa), or due to seasonal variation in the position of the oxic-anoxic interface. It has been hypothesized that MTB accumulated high polyP contents under oxic conditions, and released phosphate under anoxic conditions triggered by sulfide (Rivas-Lamelo et al., 2017; Schulz-Vogt et al., 2019).

#### 3.2.3. Potential mechanisms of PHA and polyP storage

The pathway of PHA synthesis in many bacteria involves enzymes of β-ketothiolase (PhaA), acetoacetyl CoA reductase (PhaB), and PHA polymerase (PhaC; Kessler and Witholt, 2001; García-Torreiro et al., 2016). Firstly, PhaA converts two molecules of acetyl-CoA to a molecule of acetoacetyl-CoA. The formed acetoacetyl-CoA is then stereoselectively reduced to form (R)-3-hydroxybutyryl-CoA by PhaB using NADH as the electron donor in most species. PhaC is responsible of PHA polymerization. The degradation of PHA is catalyzed by PHA depolymerase (PhaZ), which is able to hydrolyze amorphous native PHA granules yielding PHA monomers as final products (Kessler and Witholt, 2001; Liu et al., 2008; García-Torreiro et al., 2016). Similar *pha* genes have been detected in the genome of MSR-1 (Liu et al., 2008), which shares high genomic similarity with AMB-1 (Raschdorf et al., 2014). In a recent work, genomic excision of the *phbCAB* operon in MSR-1 was shown to eliminate the production of PHA granules (Raschdorf et al., 2014). In addition, presumptive magnetosome protein Mms16, associated with isolated magnetosomes from two *Magnetospirillum* strains (AMB-1 and MSR-1), is a PHB granule-bound protein (phasin) and acts *in vitro* as an activator of PHB hydrolysis (Schultheiss et al., 2005). There is however limited knowledge of the regulation of PHA metabolism in MTB at enzymatic and transcriptional level. Based on the metabolic response of other bacterial strains (e.g., *Halomonas*, *Pseudomonas* and *Azotobacter*) under nutrient deprivation, regulation of PHA metabolism might occur at different levels: activation of *pha* gene expression (i) by specific environmental signals, like nutrient starvation or (ii) by specific cell components or metabolic intermediates; (iii) inhibition of metabolic enzymes of competing pathways and consequently enrichment of required intermediates for PHA synthesis; or (iv) a combination of those (Kessler and Witholt, 2001; Castillo et al., 2013; García-Torreiro et al., 2016). Considering the higher AMB-1 growth rates and carbon consumption rates under oxic conditions (Figure 1; Supplementary Figure S2), enhanced carbon metabolism with high conversion of carbon to acetyl-CoA might result in more carbon fluxes spilled over into PHA synthesis (Castillo et al., 2013; García-Torreiro et al., 2016). Alternatively, the transition of anoxic or oxic condition might trigger the PHB synthesis by activating PHA gene expression or increasing the NADH/NAD^+^ ratio, where high NADH concentrations would inhibit citrate synthase and isocitrate dehydrogenase leading to the accumulation of acetyl-CoA for PHA synthesis (Senior et al., 1972; Senior and Dawes, 1973; Ling et al., 2018; Li et al., 2022). The exact mechanisms of O_2_ mediated PHA synthesis in MTB call for further investigations.

Previous full genome studies indicated the presence of polyP kinases (Ppk1 and Ppk2), exophosphatases (Ppx) or phosphate regulon (Pho) in MTB, including AMB-1, MSR-1, *Magnetospirillum magnetotacticum* MS-1, MC-1 and *Magnetofaba australis* strain IT-1, resulting in the potential ability of MTB to synthesis and degrade polyP granules (Zhang et al., 2002; Rao et al., 2009; Schübbe et al., 2009; Araujo et al., 2016; Zhou et al., 2017; Koziaeva et al., 2019). Pho controls phosphate uptake, and Ppk1 reversibly catalyzes the formation of polyP, whereas Ppk2 and Ppx degrade polyP to produce ATP or phosphate (Rao et al., 2009; Schübbe et al., 2009). Besides, PolyP:AMP phosphotransferase (Pap) is a class II Ppk2, which can transfer the terminal phosphate residue from poly-P to AMP, producing ADP. For example, bacterial polyP inclusions in MTB affiliated with the genus *Magnetococcus* were found to contribute substantially to the phosphorus peak observed at the lower boundary of the suboxic zone of the Black Sea (Schulz-Vogt et al., 2019). The phosphorus maximum correlated with an increase in gene expression of *ppk* by several groups of bacteria including those of the family *Magnetococcaceae*, suggesting active bacterial polyP degradation (Schulz-Vogt et al., 2019). MTB were therefore proposed to shuttle up and down within the suboxic zone, scavenging phosphate at the upper of the suboxic zone and releasing it at the lower boundary. This is consistent with the results in our batch tests: when the growth of AMB-1 resumed under oxic conditions, the biosynthesis of PHA and polyP was promoted, which could act as storage compounds for energy and carbon needed for maintenance of metabolism and synthesis of cellular metabolites under anoxic conditions.

#### 3.2.4. Possible link between PHA, polyP and magnetosome biomineralization

As homopolymers with unique molecular characteristics, both polyP and PHA (especially PHB) can assist to regulate internal ion concentrations by serving as vehicles for selective transport of ions across membranes (Reusch, 2000). PolyP is often correlated with PHB, where PHB solvates (dissolves) cations, and associates with polyP to form selective ion channels across plasma membranes (Reusch, 2000). In our work, we observed remarkable accumulations of carbon-and phosphorus-rich granules together with fewer magnetosomes (per cell) under oxic incubation periods (Figures 5, 6; Supplementary Figure S6). Since PHA formation diverts cellular resources from growth, high levels of PHA might hinder magnetosome preparation and synthesis (Fernández-Castané et al., 2017). Our results are in agreement with recent works regarding the energy competition between the formation of PHA and magnetosomes, where magnetosome production was negatively correlated with PHA formation (Raschdorf et al., 2014; Fernández-Castané et al., 2017, 2018). Furthermore, phosphate metabolism may be associated with magnetosome biosynthesis. It has been proposed that magnetite formation in MTB proceeds from the storage of iron in the form of phosphate-rich ferric hydroxide (FeP), which supposedly transforms to a transient and short-lived ferrihydrite (Fe_2_O_3_·nH_2_O) followed by the reduction to form the final magnetite mineral (Baumgartner et al., 2013). However, considering the relatively small size of magnetosomes [(1.7 ± 0.84) × 10^−3^ μm^2^], it appears difficult to follow the release of PO_4_^3−^ into medium after the separation from FeP.

### 3.3. Environmental relevance

Our results revealed strong effects of O_2_ on intracellular storage of PHA and polyP in *Magnetospirillum magneticum* strain AMB-1, indicating a tight link between oxygen, carbon and phosphorus metabolism in MTB. As a group of prokaryotes that appears to be depending on the presence of an oxic-anoxic interface in sediments or water columns, MTB can be considered as gradient organisms, shuttling with the help of magnetotaxis between oxygen-deficient and anoxic zones for oxidized and reduced (often reduced sulfur species) chemical compounds. Intracellular storage of carbon and phosphorus can facilitate efficient energy acquisition for cell metabolism and growth in anoxic environments. The intracellular storage ability of various chemical elements suggests that MTB show a great potential for biogeochemical carbon, phosphorus and iron cycling across the redox interface. Moreover, the transport of phosphorus from surface waters into deeper layers by MTB could have important implications for the retention of phosphorus in the anoxic layers and prevention of phosphorus eutrophication in surface waters. In practice, the high accumulation of PHA and polyP in MTB would enable them to be critical actors for removing excess carbon and phosphorus, and offer a promising alternative for PHA and phosphorus recovery from impaired water. The recovered polyP can be applied as new sustainable sources of phosphorus to maintain modern food production, while PHAs are a family of biodegradable polymers with promising applications for agricultural, medical and pharmaceutical industries.

## 4. Conclusion

The present study examined the accumulation and release pattern of PHA and polyP inclusions in well-studied MTB *Magnetospirillum magneticum* strain AMB-1 under manipulated redox conditions. AMB-1 growth was enhanced in the presence of O_2_, while anoxic conditions resulted in stagnated growth or a net loss of cells. Apparently fewer magnetosomes (per cell) were formed after long anoxic periods, compared to continuous oxic incubations, indicating that O_2_ likely served as regulatory signal for metabolic induction of the biomineralization of magnetosomes. Apart from magnetosomes, STEM images revealed two additional types of intercellular granules highly rich in carbon and phosphorus in AMB-1 cells, which were further interpreted as PHA and polyP based on chemical and EDX analysis. Oxygen significantly affected PHA and polyP granules, as they accounted for up to 47 ± 23% and 5.1 ± 1.7% of the cytoplasmic space, respectively, during continuous oxic conditions, while granules disappeared in anoxic incubations. Consistently, PHB and PHV accounted for 0.59 ± 0.66% and 0.0033 ± 0.0088% of dry cell weight under anoxic conditions, respectively, while the values increased up to 9.9 and 0.33% of dry cell weight under oxic conditions. Furthermore, the potential mechanisms underlying PHA and polyP storage in AMB-1 were proposed. The results advance the understanding of intracellular storage ability of MTB, and suggest their great potential for biogeochemical carbon, phosphorus and iron cycling across the redox interface.

## Data availability statement

The original contributions presented in the study are included in the article/Supplementary material, further inquiries can be directed to the corresponding author.

## Author contributions

QS: conceptualization, methodology, formal analysis, original draft preparation, and revision. DB: resources, methodology, and validation. MJ: conceptualization, methodology, writing, revision, supervision, and funding acquisition. All authors contributed to the article and approved the submitted version.

## Funding

This work was supported by a research grant (00023110) from VILLUM FONDEN.

## Conflict of interest

The authors declare that the research was conducted in the absence of any commercial or financial relationships that could be construed as a potential conflict of interest.

## Publisher’s note

All claims expressed in this article are solely those of the authors and do not necessarily represent those of their affiliated organizations, or those of the publisher, the editors and the reviewers. Any product that may be evaluated in this article, or claim that may be made by its manufacturer, is not guaranteed or endorsed by the publisher.
