# Corrigendum: Effect of oxic and anoxic conditions on intracellular storage of polyhydroxyalkanoate and polyphosphate in *Magnetospirillum magneticum* strain AMB-1

**DOI:** 10.3389/fmicb.2023.1244339

**Published:** 2023-07-12

**Authors:** Qingxian Su, Henrik Rasmus Andersen, Dennis A. Bazylinski, Marlene Mark Jensen

**Affiliations:** ^1^Department of Environmental and Resource Engineering, Technical University of Denmark, Lyngby, Denmark; ^2^School of Life Sciences, University of Nevada at Las Vegas, Las Vegas, NV, United States

**Keywords:** magnetotactic bacteria, oxygen, polyhydroxyalkanoate, polyphosphate, intracellular inclusion

In the published article, there was an error in the author list, and author “Henrik Rasmus Andersen” was erroneously excluded. The corrected author list appears below.

“Qingxian Su, Henrik Rasmus Andersen, Dennis A. Bazylinski and Marlene Mark Jensen.”

The authors apologize for this error and state that this does not change the scientific conclusions of the article in any way. The original article has been updated.

