# Supplementary material for: Effect of oxic and anoxic conditions on intracellular storage of polyhydroxyalkanoate and polyphosphate in Magnetospirillum magneticum strain AMB-1
Source: Front Microbiol. 2023 Jun 15;14:1203805. doi: 10.3389/fmicb.2023.1203805 (PMC10310966; doi:10.3389/fmicb.2023.1203805)
Supplement: Supplementary file 1 [file Data_Sheet_1.PDF]

Supplementary Material

Effect of oxic and anoxic conditions on intracellular storage of polyhydroxyalkanoate and polyphosphate in *Magnetospirillum magneticum* strain AMB-1

Qingxian Su[1], Dennis A. Bazylinski[2], Marlene Mark Jensen[1]*

[1]Department of Environmental and Resource Engineering, Technical University of Denmark, 2800 Lyngby, Denmark
[2]School of Life Sciences, University of Nevada at Las Vegas, Nevada 89154-4004, United States

*** Correspondence:**
Marlene Mark Jensen
mmaj@dtu.dk

1 Supplementary Table

Supplementary Table S1 Summary of oxygen conditions in different incubations. Oxygen concentrations were controlled at 5.1 ± 1.2 µM during oxic conditions (n = 50), while concentrations were below the detection limit of the oxygen sensors (~0.0052 µM) during anoxic conditions.

<table>
<tr><th>Day</th><th>O20</th><th>A12O8</th><th>O7A5O8</th><th>O3A3O3A3O8</th></tr>
<tr><td>1</td><td rowspan="20">oxic</td><td rowspan="12">anoxic</td><td rowspan="7">oxic</td><td rowspan="3">oxic</td></tr>
<tr><td>2</td></tr>
<tr><td>3</td></tr>
<tr><td>4</td><td rowspan="3">anoxic</td></tr>
<tr><td>5</td></tr>
<tr><td>6</td></tr>
<tr><td>7</td><td rowspan="3">oxic</td></tr>
<tr><td>8</td><td rowspan="5">anoxic</td></tr>
<tr><td>9</td></tr>
<tr><td>10</td><td rowspan="3">anoxic</td></tr>
<tr><td>11</td></tr>
<tr><td>12</td></tr>
<tr><td>13</td><td rowspan="8">oxic</td><td rowspan="8">oxic</td><td rowspan="8">oxic</td></tr>
<tr><td>14</td></tr>
<tr><td>15</td></tr>
<tr><td>16</td></tr>
<tr><td>17</td></tr>
<tr><td>18</td></tr>
<tr><td>19</td></tr>
<tr><td>20</td></tr>
</table>

2 Supplementary Figures

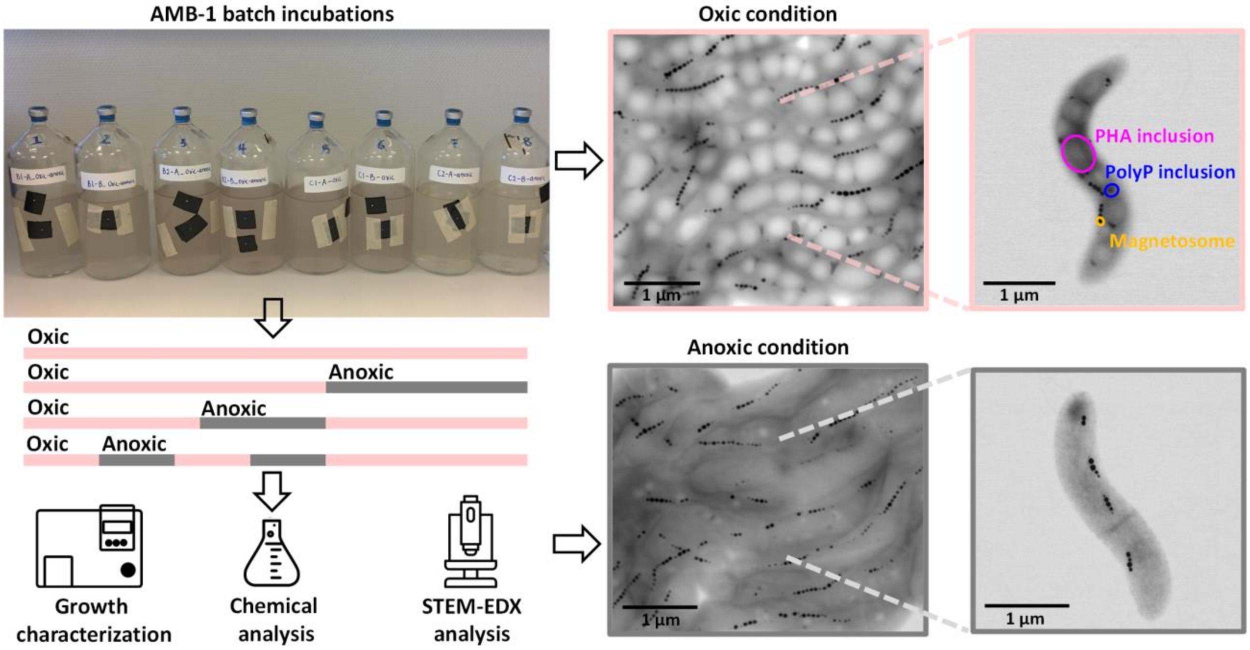

Supplementary Figure S1 Vignette of the study experimental design and methodologies.

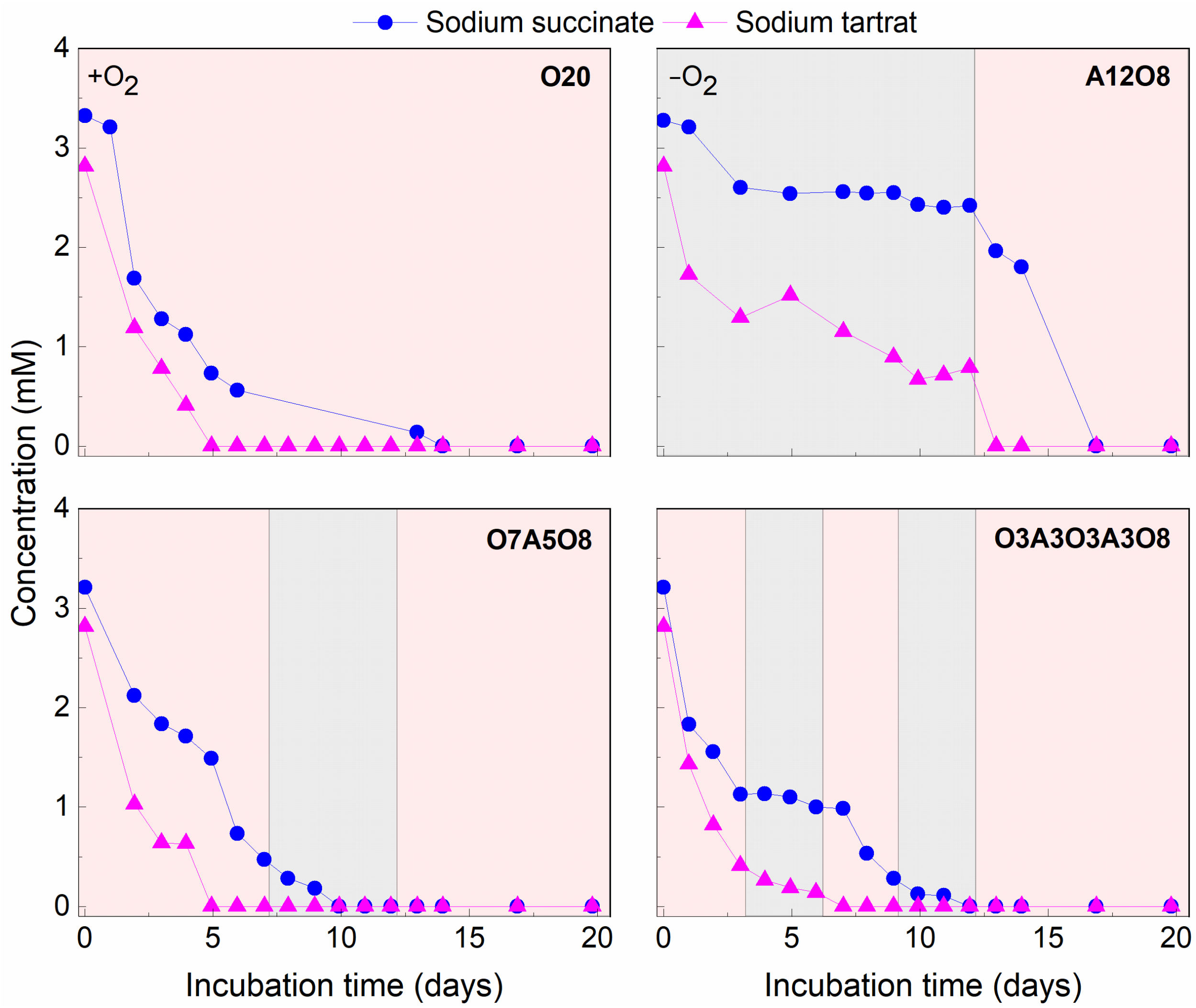

Supplementary Figure S2 Changes in bulk organic carbon concentrations (i.e., sodium succinate and sodium tartrate) during incubation period. Pink and grey colors indicate oxic and anoxic period, respectively. Concentrations of sodium acetate were below the detection limit (< 0.12 mM).

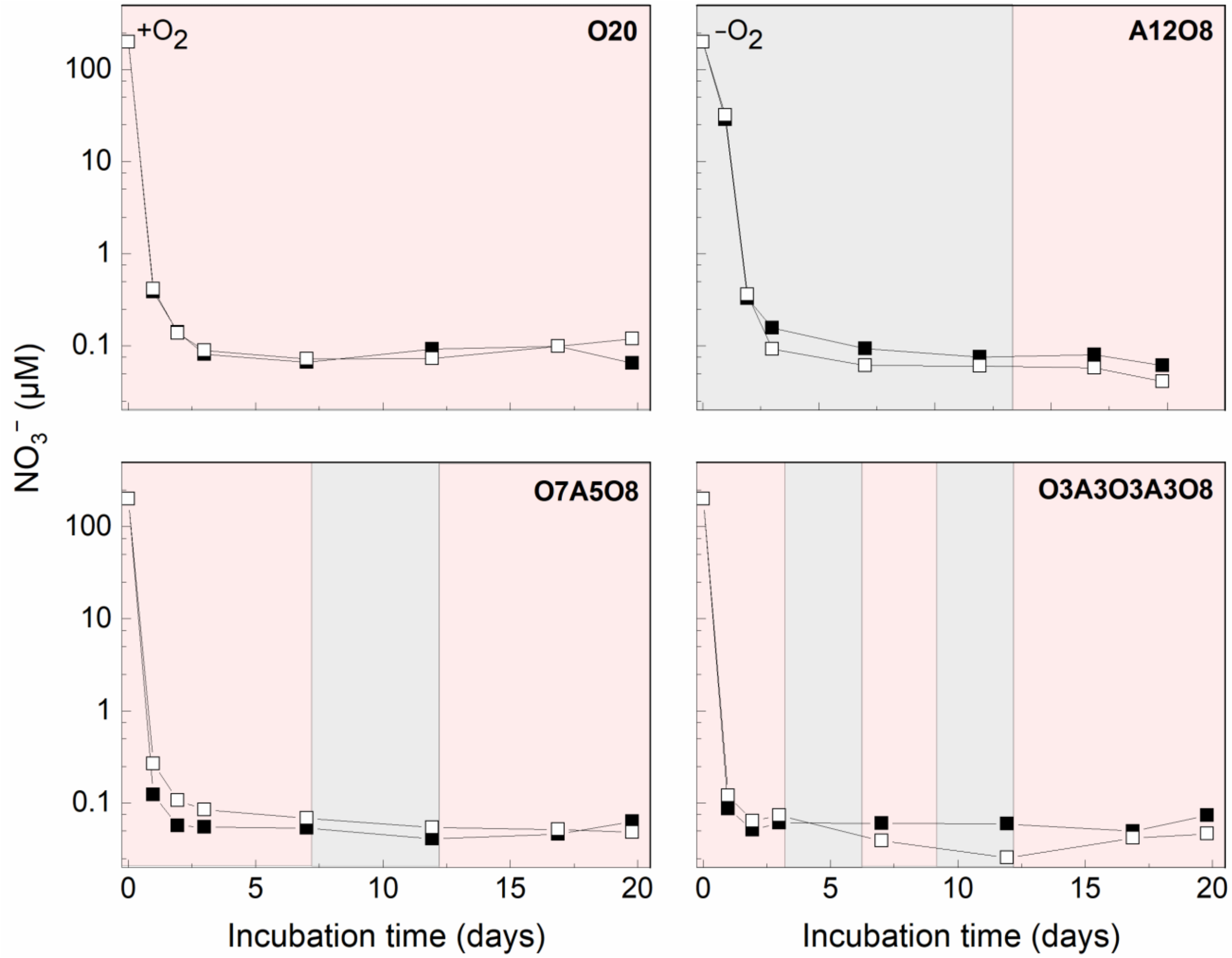

Supplementary Figure S3 Changes in bulk NO_3^- concentrations during incubation period. Pink and grey colors indicate oxic and anoxic period, respectively. Black and white squares define parallel incubations. The detection limit of NO_3^- analysis is ~ 0.065 μM.

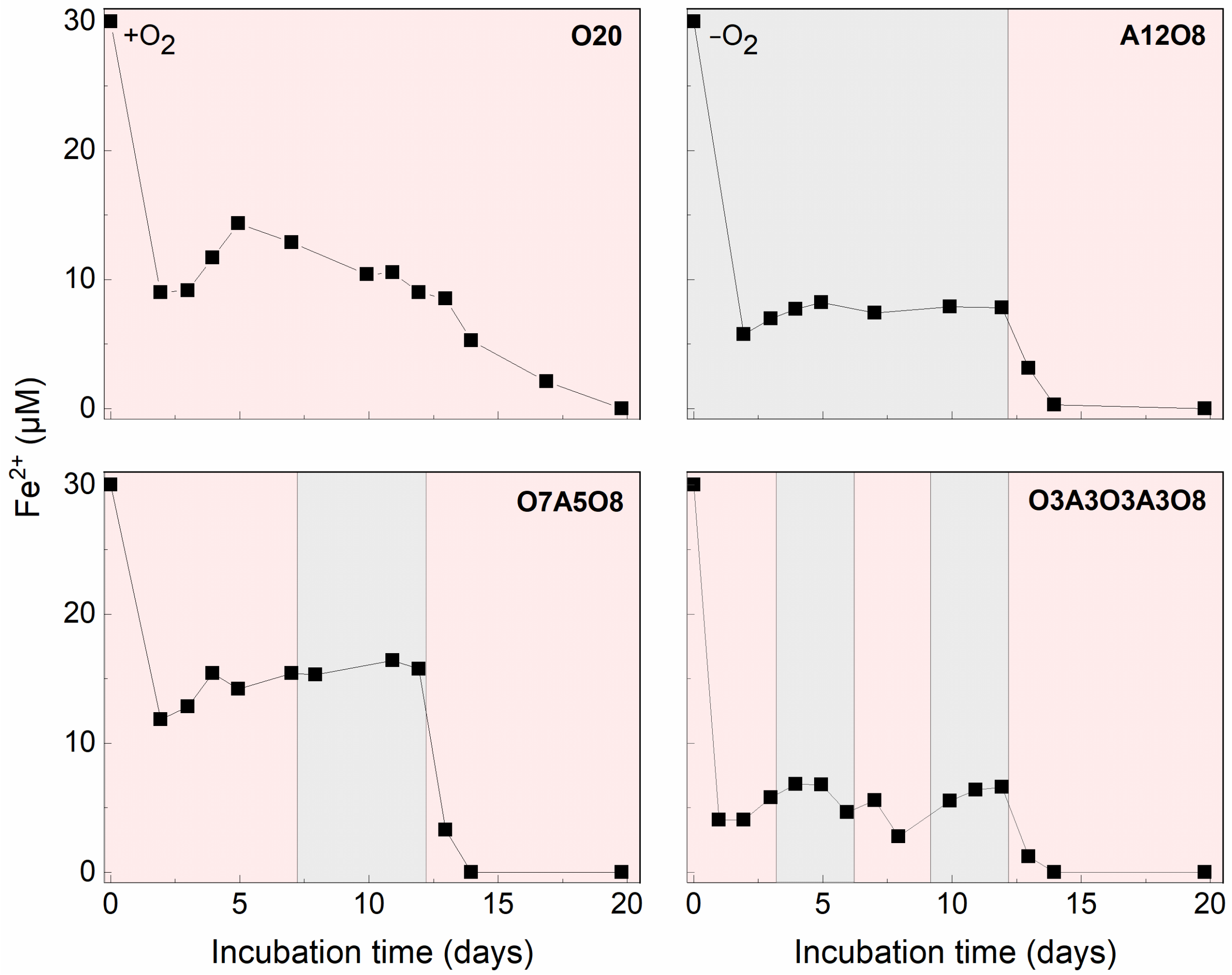

Supplementary Figure S4 Changes in bulk Fe^{2+} concentrations during incubation period. Pink and grey colors indicate oxic and anoxic period, respectively.

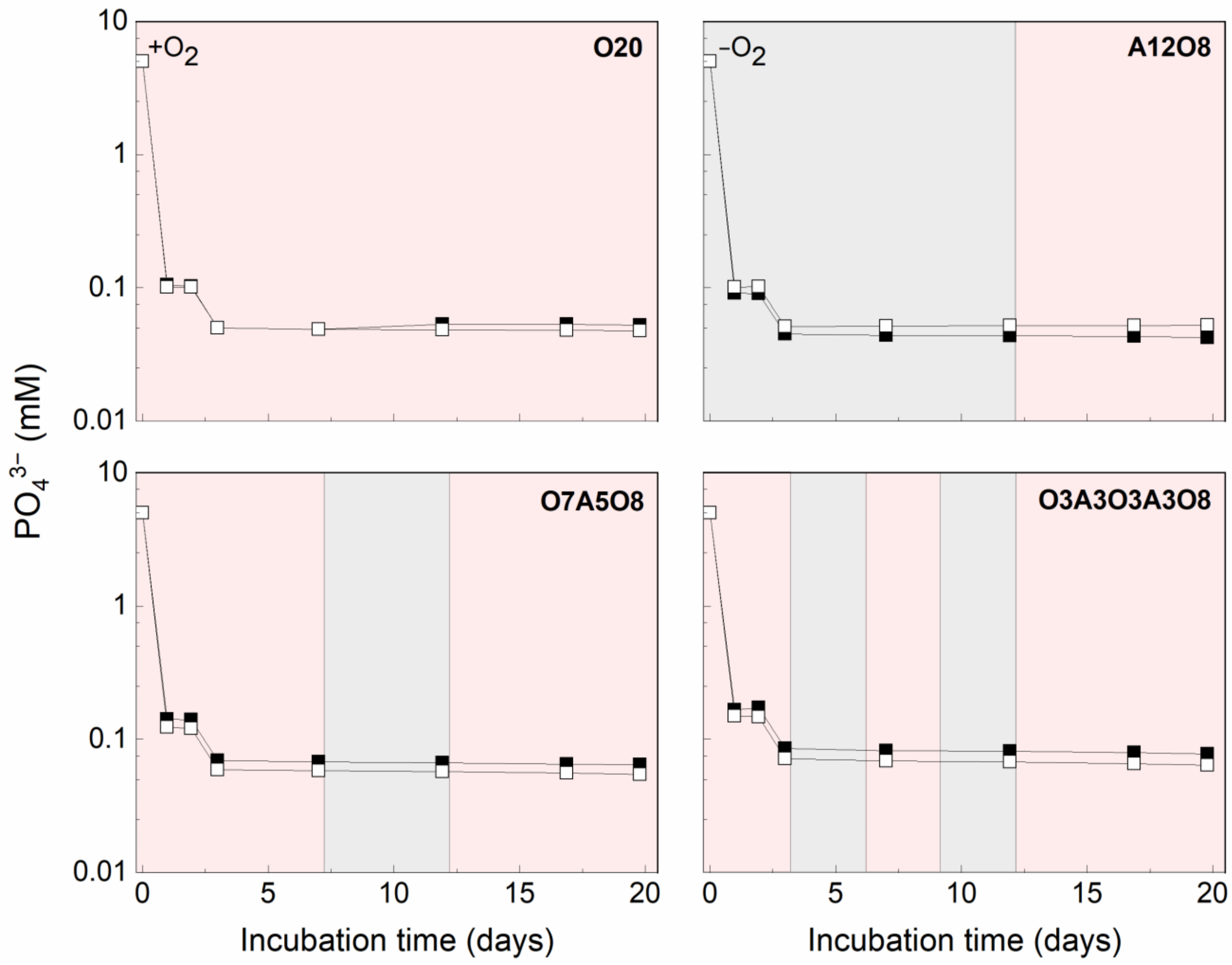

Supplementary Figure S5 Changes in PO_4^{3-} concentrations during incubation period. Pink and grey colors indicate oxic and anoxic period, respectively. Black and white squares define parallel incubations. The detection limit of PO_4^{3-} analysis is ~0.058 mM.

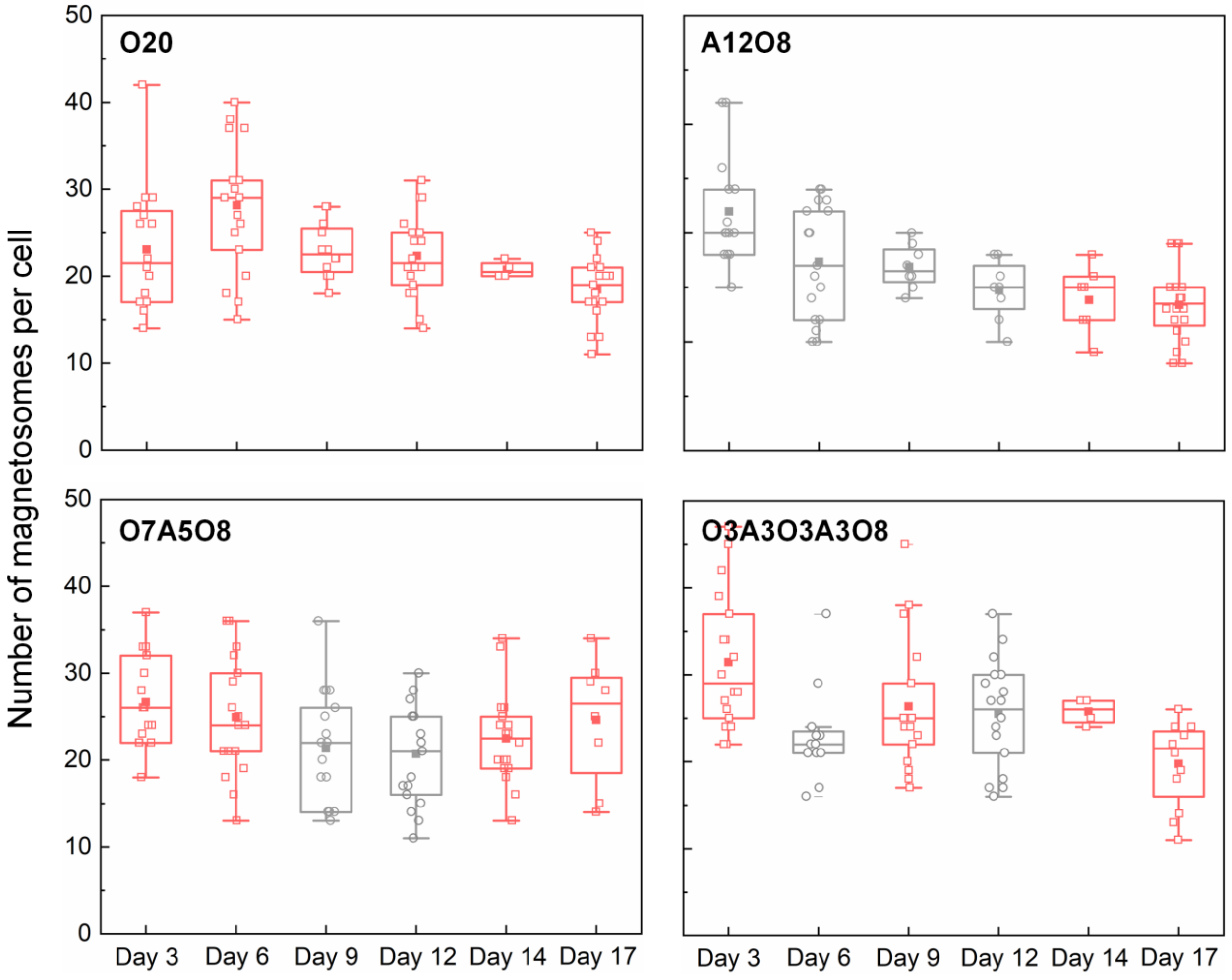

Supplementary Figure S6 Changes in the number of magnetosomes per cell during incubation period (n = 8−20 cells). Pink and grey colors indicate oxic and anoxic period, respectively.

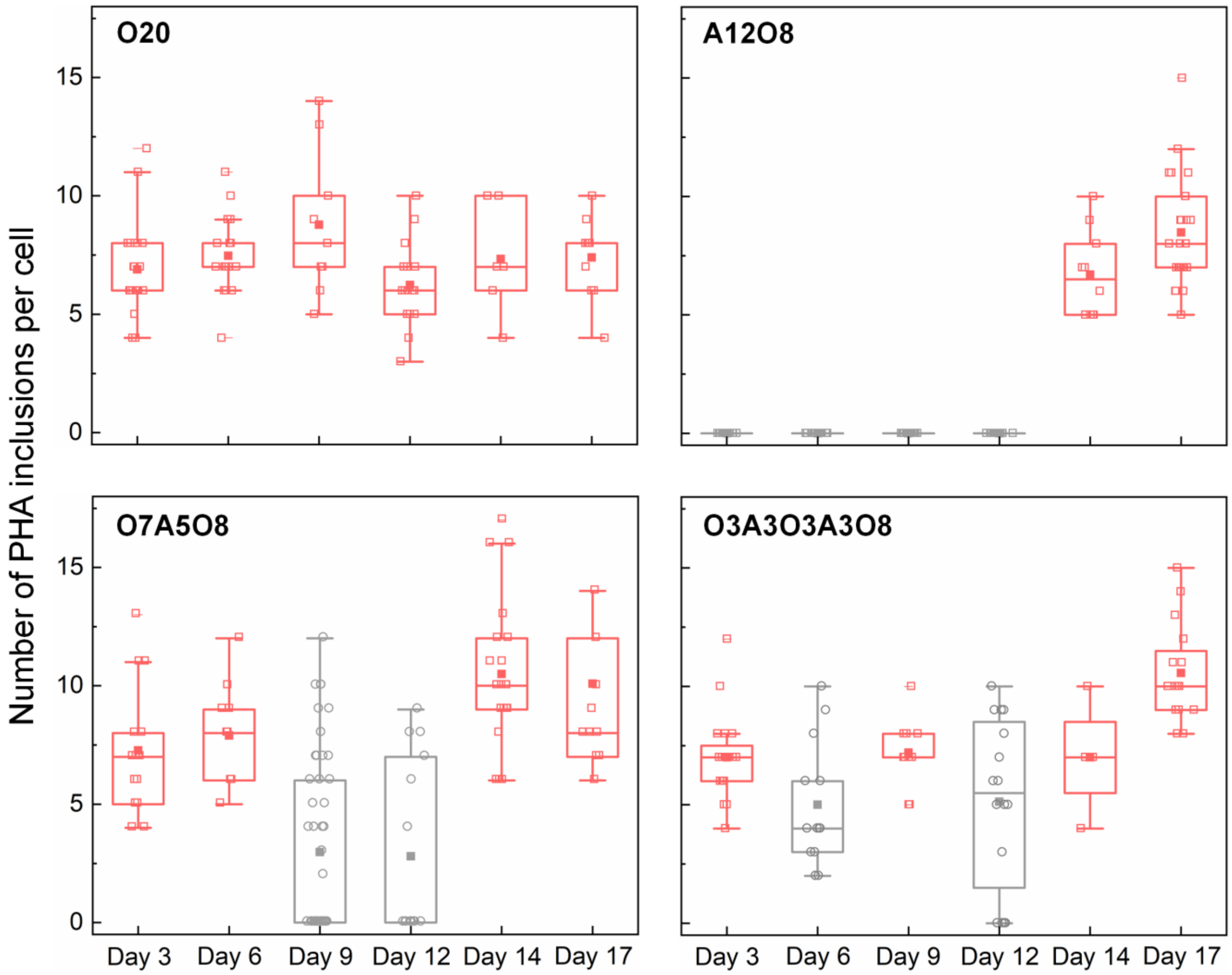

Supplementary Figure S7 Changes in the number of PHA inclusions per cell during incubation period (n = 43−212 cells). Pink and grey colors indicate oxic and anoxic period, respectively.

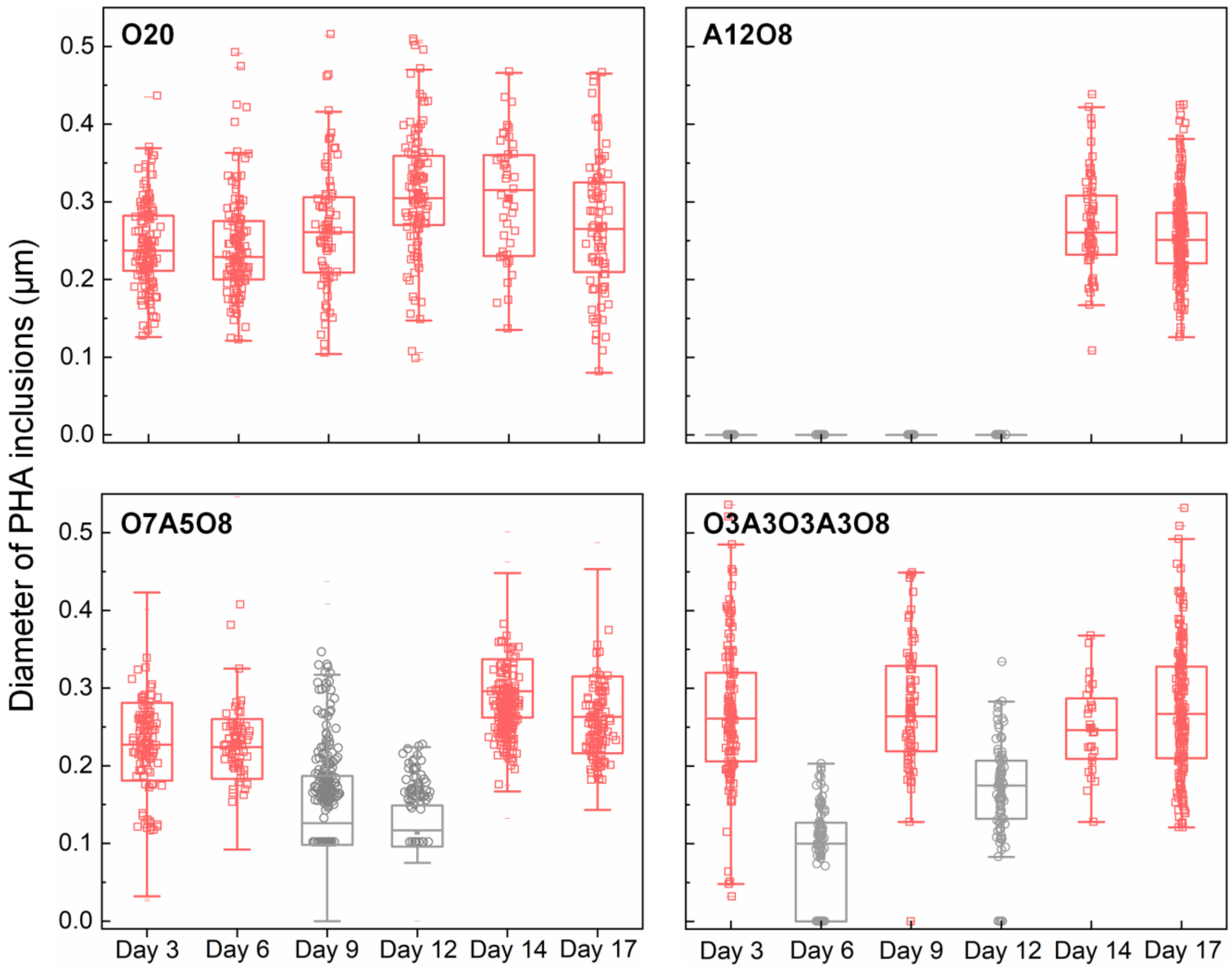

Supplementary Figure S8 Changes in the diameter of PHA inclusions during incubation period (n = 43−212 cells). Pink and grey colors indicate oxic and anoxic period, respectively.

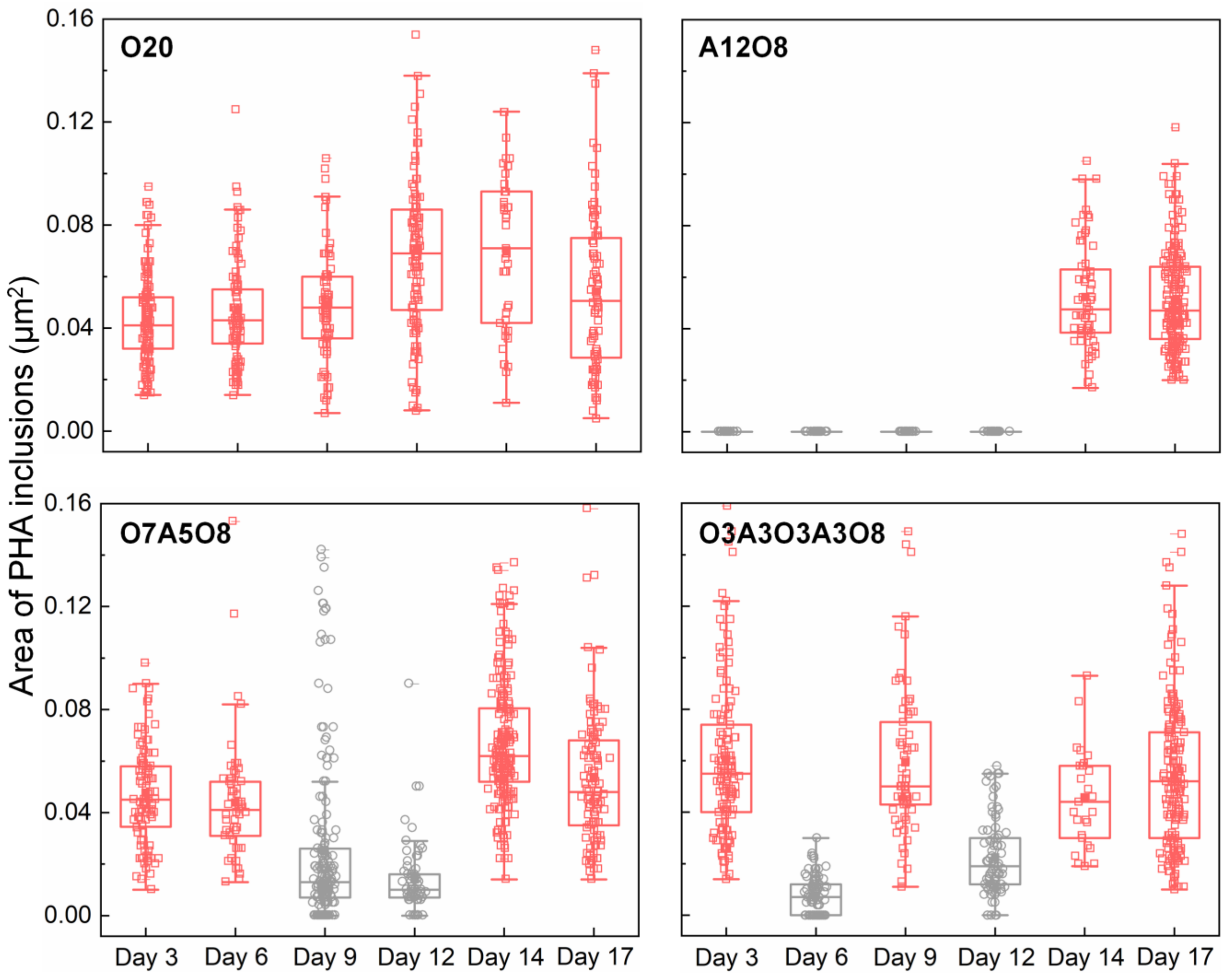

Supplementary Figure S9 Changes in the area of PHA inclusions during incubation period (n = 43−212 cells). Pink and grey colors indicate oxic and anoxic period, respectively.

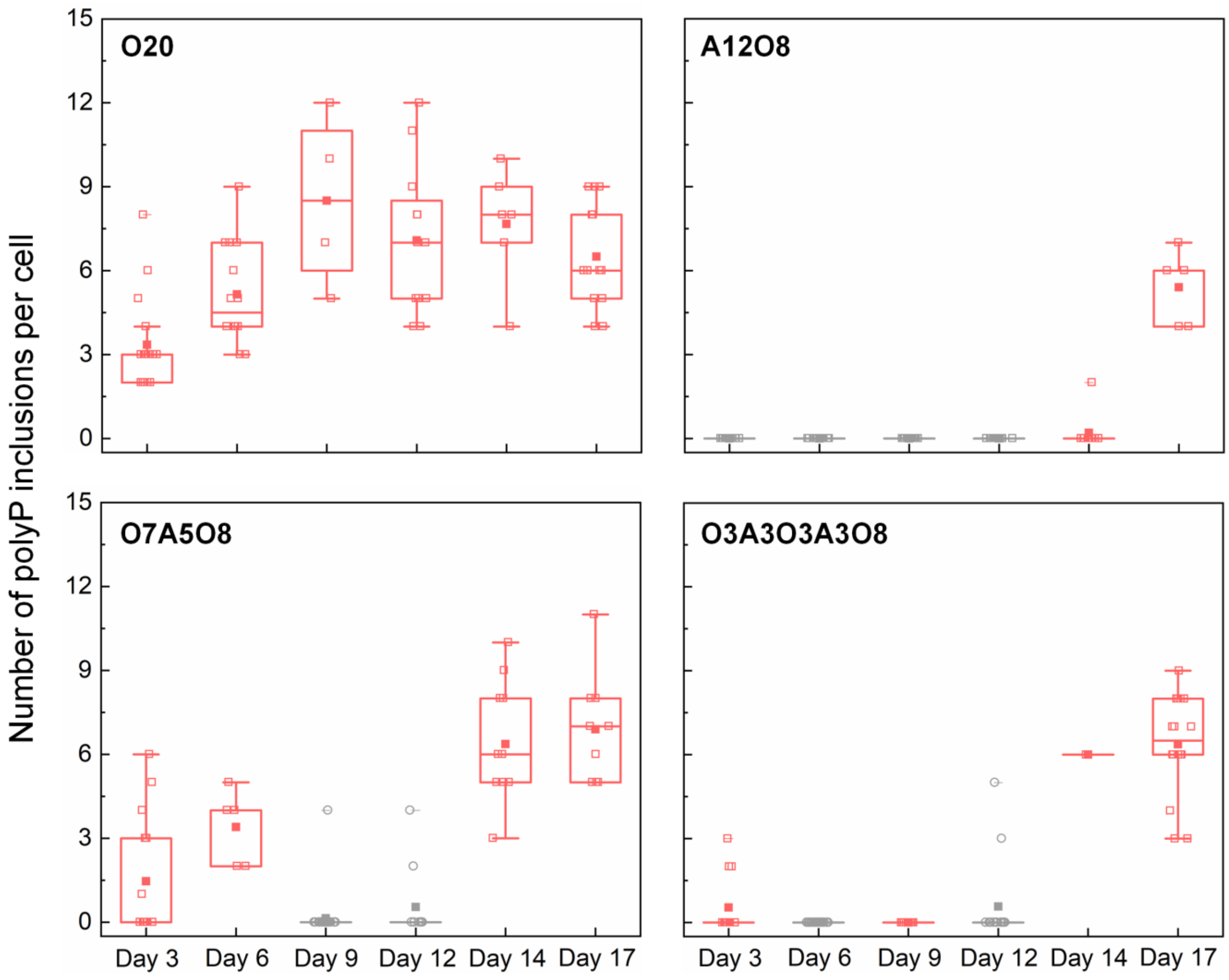

Supplementary Figure S10 Changes in the number of polyP inclusions per cell during incubation period (n = 27−114 cells). Pink and grey colors indicate oxic and anoxic period, respectively.

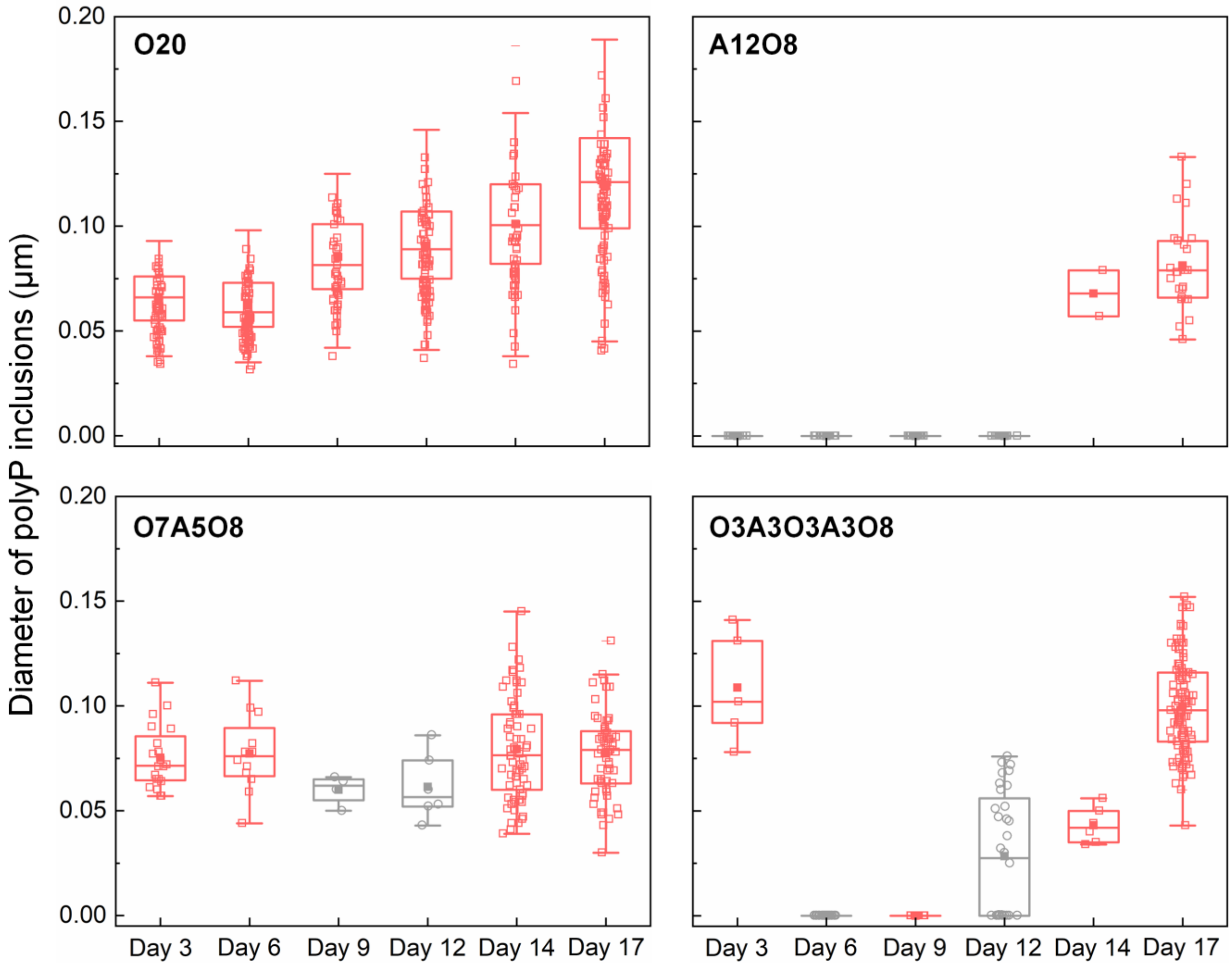

Supplementary Figure S11 Changes in the diameter of polyP inclusions during incubation period (n = 27−114 cells). Pink and grey colors indicate oxic and anoxic period, respectively.

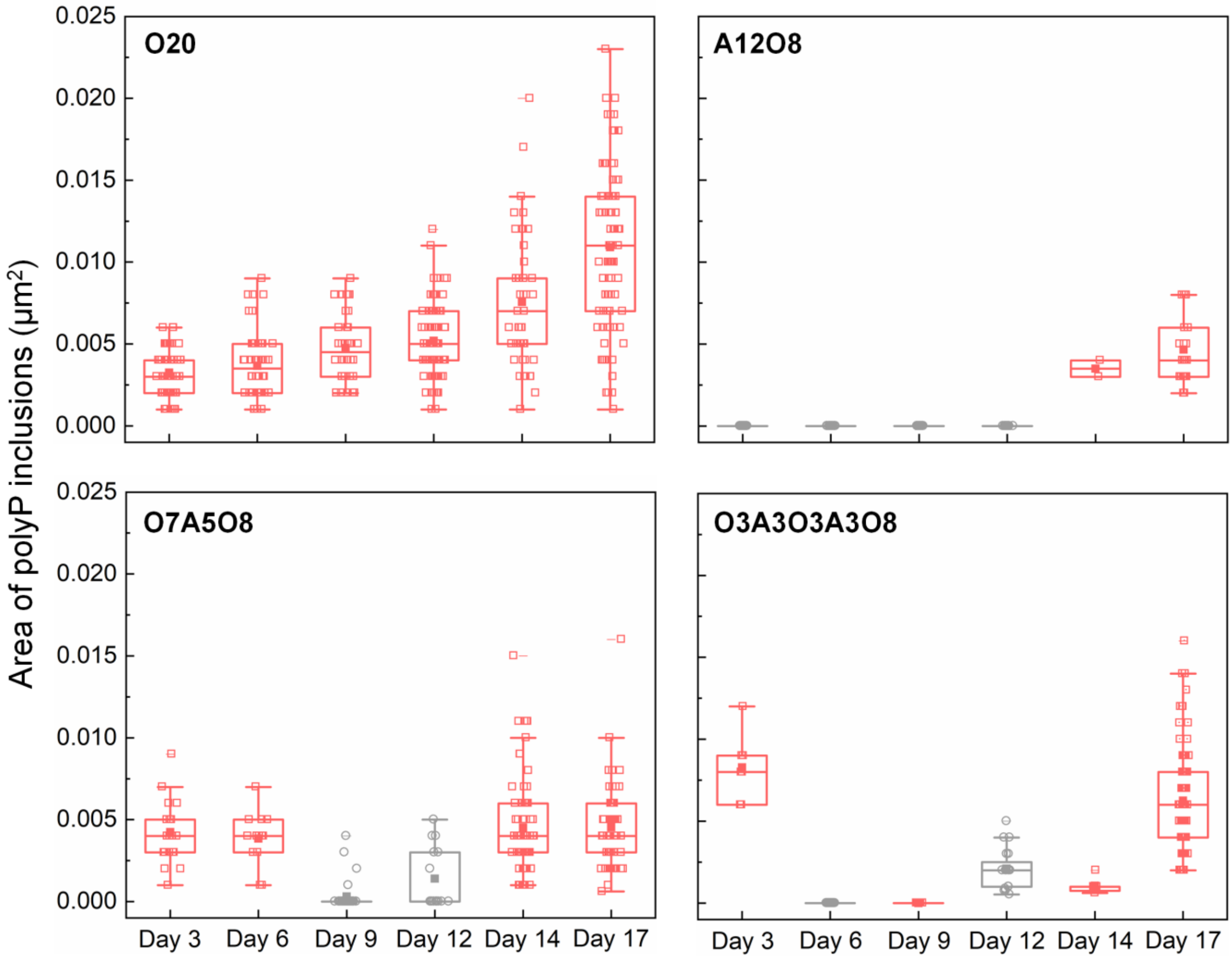

Supplementary Figure S12 Changes in the area of polyP inclusions during incubation period (n = 27−114 cells). Pink and grey colors indicate oxic and anoxic period, respectively.
